# Integrated multi-omics analyses and functional validation reveal TTK as a novel EMT activator for endometrial cancer

**DOI:** 10.1186/s12967-023-03998-8

**Published:** 2023-02-25

**Authors:** Yu Miao, Yosuke Konno, Baojin Wang, Lin Zhu, Tianyue Zhai, Kei Ihira, Noriko Kobayashi, Hidemichi Watari, Xin Jin, Junming Yue, Peixin Dong, Mingyan Fang

**Affiliations:** 1 College of Life Sciences, University of Chinese Academy of Sciences, Beijing, 100049 China; 2 BGI-Shenzhen, Shenzhen, 518083 China; 3 Department of Obstetrics and Gynecology, Hokkaido University School of Medicine, Hokkaido University, Sapporo, 060-8638 Japan; 4 Department of Gynecology and Obstetrics, Third Affiliated Hospital, Zhengzhou University, Zhengzhou, 450052 China; 5 Department of Pathology and Laboratory Medicine, University of Tennessee Health Science Center, Memphis, TN 38163 USA; 6 Center for Cancer Research, University of Tennessee Health Science Center, Memphis, TN 38163 USA; 7 BGI Research Asia-Pacific, BGI, Singapore, 138567 Singapore

**Keywords:** Cancer biomarker, Cancer diagnosis, Prognostic marker, TTK, microRNA-21, EMT, Endometrial cancer

## Abstract

**Background:**

Cancer-testis antigens (CTAs) are often expressed in tumor and testicular tissues but not in other normal tissues. To date, there has been no comprehensive study of the expression and clinical significance of CTA genes associated with endometrial cancer (EC) development. Additionally, the clinical relevance, biological role, and molecular mechanisms of the CTA gene TTK protein kinase (*TTK*) in EC are yet to be fully understood.

**Methods:**

Using bioinformatics methods, we comprehensively investigated the genomic, transcriptomic, and epigenetic changes associated with aberrant TTK overexpression in EC samples from the TCGA database. We further investigated the mechanisms of the lower survival associated with TTK dysregulation using single-cell data of EC samples from the GEO database. Cell functional assays were used to confirm the biological roles of TTK in EC cells.

**Results:**

We identified 80 CTA genes that were more abundant in EC than in normal tissues, and high expression of *TTK* was significantly linked with lower survival in EC patients. Furthermore, ROC analysis revealed that TTK could accurately distinguish stage I EC tissues from benign endometrial samples, suggesting that TTK has the potential to be a biomarker for early EC detection. We found *TTK* overexpression was more prevalent in EC patients with high-grade, advanced tumors, serous carcinoma, and *TP53* alterations. Furthermore, in EC tissue, TTK expression showed a strong positive correlation with EMT-related genes. With single-cell transcriptome data, we identified a proliferative cell subpopulation with high expression of TTK and known epithelial–mesenchymal transition (EMT)-related genes and transcription factors. When proliferative cells were grouped according to *TTK* expression levels, the overexpressed genes in the TTK^high^ group were shown to be functionally involved in the control of chemoresistance. Utilizing shRNA to repress TTK expression in EC cells resulted in substantial decreases in cell proliferation, invasion, EMT, and chemoresistance. Further research identified microRNA-21 (miR-21) as a key downstream regulator of TTK-induced EMT and chemoresistance. Finally, the TTK inhibitor AZ3146 was effective in reducing EC cell growth and invasion and enhancing the apoptosis of EC cells generated by paclitaxel.

**Conclusion:**

Our findings establish the clinical significance of TTK as a new biomarker for EC and an as-yet-unknown carcinogenic function. This present study proposes that the therapeutic targeting of TTK might provide a viable approach for the treatment of EC.

**Supplementary Information:**

The online version contains supplementary material available at 10.1186/s12967-023-03998-8.

## Introduction

Endometrial cancer (EC) is a prevalent type of cancer affecting women globally. According to statistics from 2020, there were approximately 417,000 new cases and 97,000 deaths of EC reported, ranking it as the sixth most common cancer among women [1]. Despite the favorable prognosis for patients with early-stage tumors, who have a 5-year survival rate of more than 75%, advanced EC has a poorer prognosis with a 5-year survival rate of only 20–26% for stage advanced EC patients [2]. This highlights the importance of comprehending the molecular processes of EC and identifying sensitive and specific markers for early detection, prognostic prediction, personalized treatment, and ultimately, improved patient outcomes.

Current standard first-line chemotherapy for EC relies heavily on the combination of carboplatin and paclitaxel. It can achieve remission ratios of 43–62% [3], which implies that 40–60% of individuals continue to demonstrate chemoresistance, significantly decreasing their survival. Resistance to chemotherapeutic medicines necessitates the development of novel methods for enhancing their sensitivity. Cancer cells that undergo epithelial-mesenchymal transition (EMT) acquire mesenchymal features, immune evasion, cancer stem cell maintenance, cancer metastasis, and chemoresistance [4]. Targeting EMT inducers, such as EZH2 and BMI-1, may prevent cancer metastasis and improve the effectiveness of anti-cancer drugs [4, 5].

Cancer-testis antigen (CTA) is a wide family of cancer-specific antigens that express proteins only in human germ line cells of the testis and a variety of human malignancies [6]. CTA antigens have been discovered as potential biomarkers for cancer diagnosis and treatment due to their highly immunogenic, cancer-specific expression pattern [7]. Previously, CAGE, a CTA, was shown to be expressed in EC cell lines (Ishikawa and HEC-1), but not in normal melanocyte cell lines [8]. In addition, the protein expression of another CTA HIWI was detected in EC tissues by using immunohistochemistry [9]. A systematic investigation of the expression and clinical significance of CTA genes involved in EC development is yet lacking.

TTK is a CTA that plays a critical role in driving EMT and chemoresistance in human malignancies [10, 11]. Pharmacological inhibition and genetic silencing of TTK not only reversed the EMT process, but also greatly reduced invasion in triple-negative breast cancer cells [10]. In ovarian cancer, knockdown or inhibition of TTK expression was known to drastically restrict cell growth and eliminate cisplatin resistance [11]. TTK has been identified as a poor prognostic biomarker in EC by recent investigations [12]. Although a TTK inhibitor (NTRC0066-0) has been shown to greatly reduce the growth of the EC cells [13], the detailed cellular function of TTK in EMT and the progression of EC, as well as the significance of TTK inhibitors in the treatment of EC, remain unknown.

Molecular approaches, such as microarray gene expression profiling and next-generation sequencing, have dramatically improved genome-wide analyses of tumor gene expression patterns and, as a result, have been widely used in developing new diagnostic, prognostic, and predictive biomarkers [14]. The Cancer Genome Atlas (TCGA) database has a wealth of large-scale gene expression data on gynecological malignancies. Current cancer biomarkers discovered by meta-analysis may be of little therapeutic use, perhaps due to a lack of functional validation of biomarker genes.

The key questions that need to be resolved are: (1) the expression pattern of CTA genes in EC; (2) the function of TTK in the development of EC and drug resistance; (3) the molecular mechanisms by which TTK promotes the development of cancer and chemoresistance; and (4) the discovery of effective TTK inhibitors for the treatment of EC.

In this study, we sought to comprehensively examine the expression and prognostic significance of CTA genes involved in EC development. This research also characterized the cellular functions and underlying processes of TTK in regulating cell proliferation, invasion, and paclitaxel (TX) chemoresistance. Finally, we attempted to give a theoretical basis for the future use of a combination of TX and a TTK inhibitor, AZ3146, in the treatment of EC. We discovered that several CTA genes (including *TTK*) were discovered to be overexpressed in EC tissues compared to normal tissues. TTK, as a new EC biomarker, has potential diagnostic and prognostic usefulness. We verified that TTK enhances EMT phenotypes and chemoresistance in EC cells by increasing microRNA-21 (miR-21) expression. TTK inhibition with AZ3146 increased EC cell apoptosis induced by TX. Given the finding of TTK’s crucial role in generating EMT and chemoresistance, targeting TTK may be beneficial in treating EC patients.

## Methods

### Differential gene analysis and weighted gene co-expression network analysis

Differential analysis was performed using Deseq2 [15] for RNAseq data (TCGA) and Limma for microarray data (GSE17025) using log2|fold-change| > 1 and p_adjust < 0.05 as thresholds to filter differential genes. Based on WGCNA [16] package, the top 5,000 protein-coding genes were used to construct a co-expression network by weighted correlation network analysis. After identifying co-expression modules, we identified the hub genes of the modules using thresholds of GS > 0.2 and MM > 0.8. Weighted Gene Co-expression Network Analysis (WGCNA) package [16] (R package, version 1.71) was used to construct a co-expression network based on the first 5000 protein-coding genes. The “pickSoftThreshold” function was employed to determine the optimal soft threshold for the module construction. The co-expression networks (modules) were formed using the “blockwiseModules” algorithm with a minimum size of 25, a deepSplit of 3 and a mergeCutHeight of 0.25, after ensuring that the R-squared value reached 0.85.

### Survival analysis and cox regression

The EC samples from TCGA were divided into two groups based on gene expression: high expression and low expression. This division was performed by using either the median expression of a specific gene or the median average expression of all genes in a module as the dividing line. The survival package was utilized to plot the Kaplan–Meier (KM) curves and compare the survival risk between the two risk groups. Univariate Cox proportional risk regression analysis of survival data for each differential gene between the CTA^high^ and CTA^low^ groups was performed using the coxph function of the survival (R package) with a significance threshold set at p.adjust < 0.05. Finally, 3 CTA genes with significantly different expressions related to prognosis were found. The gene expression was normalized to Transcripts Per Million (TPM) values before performing the survival analysis and cox proportional hazard regression.

### ROC analysis of the TTK gene

For the evaluation of the gene TTK's performance as a classifier, a logistic regression model was fit due to the binary classification nature of the data. A classical Receiver Operating Characteristic (ROC) analysis was performed using the “roc” function of the pROC package [17] (version 1.16.2) in R, with the “smooth” parameter set to TRUE. The performance of the classifier was measured by the size of the Area Under the Curve (AUC).

### Tumor immune estimation resource database

Differences in gene expression of TTK in pan-cancer and normal tissues were downloaded from the TIMER database (https://cistrome.shinyapps.io/timer/).

### The human protein atlas database

The protein expressions of target genes in human normal tissues and tumor tissues were validated via the Human Protein Atlas (HPA, https://www.proteinatlas.org/). Pathological images of immunohistochemical staining of target proteins and subcellular localization results can be downloaded from this website.

### Mutational spectrum analysis

Based on the somatic mutation data (files in MAF format) of ECs in TCGA, we identified the significantly mutated genes (SMG) using MutsigCV [18] with a filtering threshold of q-value < 0.1. Maftools [19] and GenVisR [20] programs were used to visualize SMGs.

### Copy number variation analysis

GISTIC2 [21] was used to determine genes with significant amplification or deletion. The parameter thresholds for fragments with amplification or deletion lengths were *p*-adjusted < 0.25. Maftools were used to visualize dramatically amplified or deleted fragments.

### Differential methylation analysis

Using |Δβ| > 0.2 and p_adjust < 0.05 as thresholds to filter differentially methylated genes, 450K methylation microarray data from EC samples in TCGA were subjected to a differential methylation analysis using CHAMP (R package, version 2.20.1) [22]. For differentially expressed genes whose promoters were differentially methylated, we performed correlation analysis using the methylation levels of the differentially methylated positions (DMPs) with the expression of the corresponding genes.

### Enrichment analysis

Functional enrichment: the clusterProfiler [23] and kobas3.0 packages were used for GSEA and KEGG enrichment analysis, respectively. Proliferative scoring for RNAseq and microarray data: the top 20 significantly highly expressed genes for proliferative cell subpopulation analysis were screened according to log2FC. Single sample gene set enrichment analysis (ssGSEA) was performed using the “gsva” function from the GSVA software [24] (version 1.38.2) in R. The parameters for the analysis were set as follows: method as ssgsea, kcdf as Gaussian, and abs.ranking as TRUE.

### Single-cell transcriptome data processing

Single-cell expression matrices of 5 EC samples were downloaded from the GEO database (GSE173682). DoulbletFinder [25] (version 2.0.3) was applied to infer and remove doublets. Cells with > 15% of mitochondrial genes or < 200 or > 6000 expressed genes were considered doublets and removed. Besides, we also removed all ribosomal genes. We used Seurat 4.0.1 [26] (resolution = 1, PC = 1:30) to downscale and cluster single-cell expression data after removing batch effects using the harmony [27] package. Cell types were defined by using SingleR and classical cell markers. The Findallmarker function was used to identify highly expressed genes in each subpopulation with the following parameters: log2|fold-change| > 0.25 and p_adj < 0.05.

### Estimation of CNV analysis and malignant epithelial cell inference

Copy number variations (CNVs) were detected using the InferCNV software [28] (version 1.6.0) in R. The raw count matrix was used as input data, and the endothelial cells were used as the reference (normal). The “run” function was used to infer CNVs in the epithelial cells, with parameters set as cutoff as 0.1, cluster_by_groups, and denoise set as TRUE.

### Cell culture

HEC-1 cells were received from the JCRB Cell Bank (Osaka, Japan), while Ishikawa cells were obtained from the American Type Culture Collection (Manassas, VA, USA). Benign endometrial epithelial cells (EM) were kindly provided by Dr. Satoru Kyo (Shimane University, Japan). These cells were maintained at 37 °C with 5% CO_2_ in DMEM/F12 media (Sigma-Aldrich, St. Louis, MO, USA) containing 10% fetal bovine serum (FBS, Thermo Fisher Scientific, Carlsbad, CA, USA) and 100 g/ml Normocin (Invivogen, San Diego, CA, USA).

### RNA extraction and real-time quantitative PCR

The TRIzol reagent (Thermo Fisher Scientific) was used to isolate RNA, and the concentration and purity of the samples were evaluated using the NanoDrop ND-2000. Reverse transcription was performed on RNA using a Reverse Transcription Kit (Takara, Shiga, Japan). PrimeScript RT-PCR kit (Takara) was used in the qPCR experiments. The primers (as stated below) were retrieved from the PrimerBank database: human *TTK*, forward (F) 5′-GTGGAGCAGTACCACTAGAAATG-3′; reverse (R): 5′-CCCAAGTGAACCGGAAAATGA-3′; human *CK-18*, F: 5′-GGCATCCAGAACGAGAAGGAG-3′; R: 5′-ATTGTCCACAGTATTTGCGAAGA-3′; human *BMI-1*, F: 5′-CCACCTGATGTGTGTGCTTTG-3′; R: 5′-TTCAGTAGTGGTCTGGTCTTGT-3′; human *EZH2*, F: 5′-AATCAGAGTACATGCGACTGAGA-3′; R: 5′-GCTGTATCCTTCGCTGTTTCC-3′; human *ZO-1*, F: 5′-CAACATACAGTGACGCTTCACA-3′; R: 5′-CACTATTGACGTTTCCCCACTC-3′; human *Vimentin* (*VIM*), F: 5′-AGTCCACTGAGTACCGGAGAC-3′; R: 5′-CATTTCACGCATCTGGCGTTC-3′; human *Fibronectin* (FN), F: 5′-CGGTGGCTGTCAGTCAAAG-3′; R: 5′-AAACCTCGGCTTCCTCCATAA-3′; human *β-actin*, F: 5′-CATGTACGTTGCTATCCAGGC-3′, R: 5′-CTCCTTAATGTCACGCACGAT-3′. The expression of genes was normalized to *β*-actin mRNA expression. The NCode EXPRESS SYBR GreenER microRNA qRT-PCR Kit (Thermo Fisher Scientific) was used to verify miRNA expression with miR-21 forward primer and universal qRT-PCR Primer (Thermo Fisher Scientific). The expression of miR-21 was normalized to the levels of U6.

### Cell transfection

2 μg of TTK overexpression vector and the corresponding control vector (Origene, Rockville, MD, USA) or 2 μg of TTK-targeting shRNAs (sc-36758-SH, Santa Cruz Biotechnology) and a control shRNA (sc-108060, Santa Cruz Biotechnology) were used per well for 12-well plates to transfect Ishikawa and HEC-1 cells using the Lipofectamine 3000 transfection reagent (Thermo Fisher Scientific). Thermo Fisher Scientific provided the miR-21 mimic/inhibitor (30 nM) and the control mimic/inhibitor (30 nM), which were transfected into EC cells using the Lipofectamine 3000 transfection reagent.

### Western blotting

EC cells were lysed using M-Per Mammalian Protein Extraction Reagent (Pierce, Rockford, IL, USA). By 10% SDS-polyacrylamide gel electrophoresis, protein samples (20 μg) were separated and transferred to a PVDF membrane (Millipore, Bedford, MD, USA). The membrane was incubated for 2 h overnight at 4 °C with a primary antibody against human TTK (1:1000; Cell Signaling Technology, Beverly, MA, USA) or β-actin (1:5000, Cell Signaling Technology), followed by 1 h at room temperature with a secondary antibody. The band was evaluated using enhanced chemiluminescence detection reagents (Pierce, Rockford, IL, USA).

### Functional assays

Cell proliferation was determined using the Cell Counting Kit-8 (CCK-8) assay (Dojindo, Japan). After 72 h, 10 µl of CCK-8 reagent was applied to the experimental cell-containing wells and incubated for up to 4 h before determining the absorbance at 450 nm. The cell invasion assay was carried out in a Matrigel-coated transwell chamber with a pore size of 8 µm (Corning Costar, Lowell, CA, USA). The bottom compartment was filled with growth medium supplemented with 10% FBS, and the transwell inserts were seeded with 5 × 10^4^ cells suspended in serum-free culture media. Cells were cultured at 37 °C for 24 h before being fixed in paraformaldehyde and stained with crystal violet. Using an upright Nikon microscope, invaded cells were observed and photographed.

### Cytotoxicity of the TTK inhibitor AZ3146

In 96-well plates, 5000 EC cells per well were seeded and left to adhere overnight. These cells were treated for an additional 24 h with or without AZ3146 (sc-361114, Santa Cruz Biotechnology) at doses ranging from 0 to 90 nM. MTT assays (BioAssay Systems, Hayward, CA, USA) were used to assess cell viability.

### Investigation of cell apoptosis

The Caspase-Glo 3/7 Assay kit (Promega, Madison, WI, USA) was used to quantify apoptotic activity in EC cells. EC cells were allowed 24 h to adhere to the plate. In 100 µl of medium, HEC-1 and Ishikawa cells were treated with TX as indicated in the presence of AZ3146 or DMSO for 24 h. 100 µl of luminescence substrate for Caspase-3/7 was then added to each well and stirred gently. After 90 min of incubation at room temperature and in the dark, luminescence was measured using a microplate reader (Thermo Fisher Scientific).

### Statistical analysis

Using the Kaplan–Meier approach, survival curves were created and analyzed using the log-rank tests. Two-tailed Student’s *t*-tests and Wilcoxon tests were used when appropriate. The error bars show the standard error of the mean collected from at least three different experiments. When *P*-values were less than 0.05, the results were considered significant. All graphs were plotted based on R 4.0.4.

## Results

### Identification of EC-associated CTA genes

**Fig. 1:**
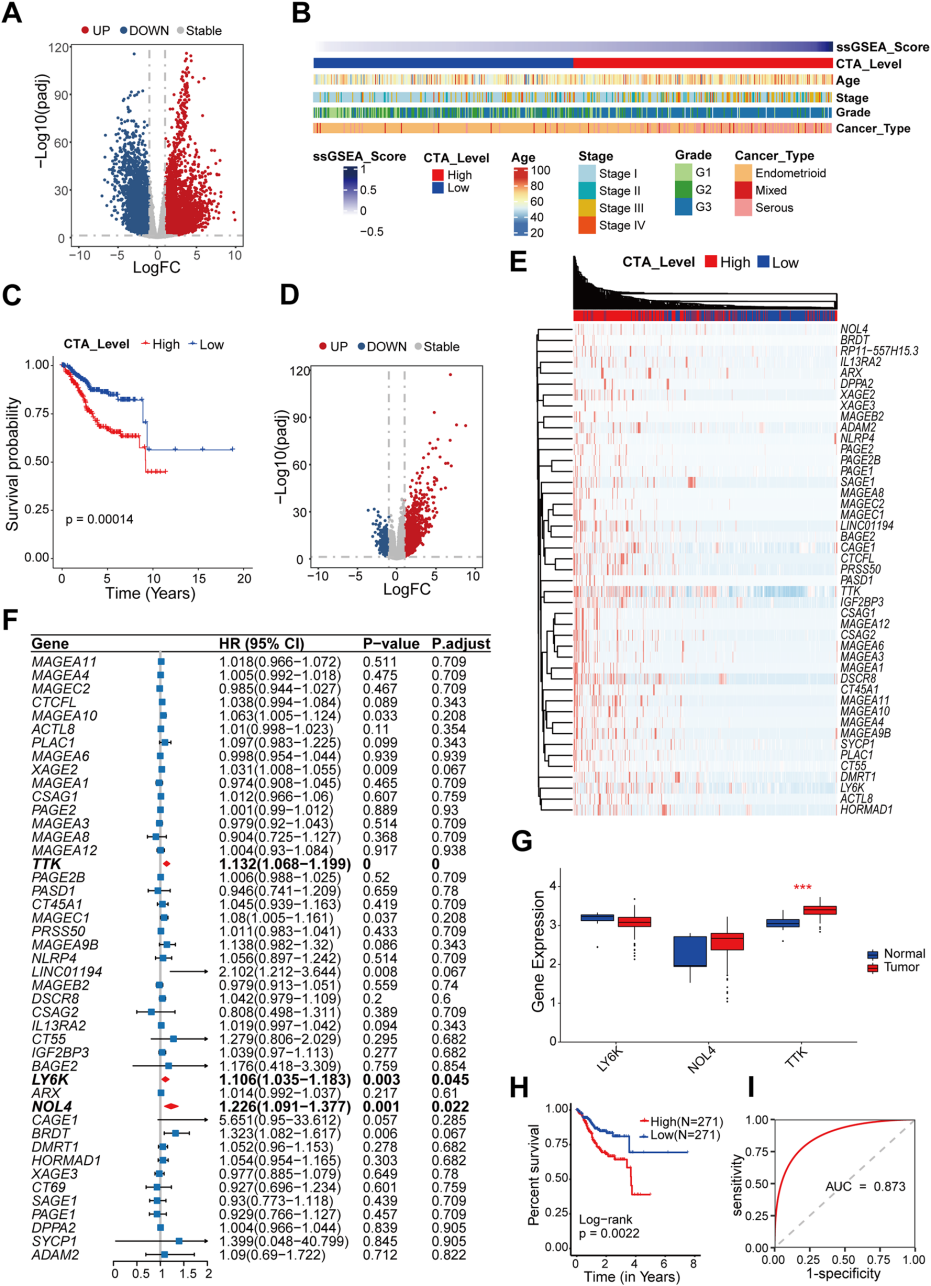
Identification of EC-associated CTA genes. **A** Differential genes between tumor and para-cancer; Red dots indicate genes significantly overexpressed in the tumor, and blue dots indicate genes significantly downregulated in para-cancer. The screening threshold is (log2|fold-change| > 1 and *p-*adjust < 0.05). The horizontal gray dashed line means *p-*adjust = 0.05, and the vertical gray dashed line means log2|fold-change | = 1. **B** ssGSEA enrichment analysis based on CTA genes that upregulated in EC showed a strong correlation between CTA enrichment scores and clinical features. **C** Results of survival analysis based on CTA enrichment scores. A two-sided log-rank test is used to compare patient survival between the two groups. **D** Differential genes between the CTA^high^ group and CTA^low^ group; red dots indicate genes significantly overexpressed in the CTA^high^ group, and blue dots indicate genes significantly overexpressed in the CTA^low^ group; the screening threshold is (log2|fold-change| > 1 and p-adjust < 0.05). The horizontal gray dashed line means *p*-adjust = 0.05, and the vertical gray dashed line means log2|fold-change| = 1. **E** Heat map shows the CTA genes that are co-upregulated in the CTA^high^ group and EC (**F**) Univariate cox regression analysis of the CTA gene in E plot. **G** Differential gene expression of three genes (*TTK*, *LY6K*, *NOL4*) in stage 1 EC tissues (GEO database) and benign samples (GSE17025). ***P < 0.001, by Wilcoxon tests. **H** Survival analysis of TTK based on EC gene expression profile from TCGA database. A two-sided log-rank test is used to compare patient survival between the two groups. **I** *TTK* expression has potential diagnostic significance in distinguishing early-stage EC tissues from benign tissues. The ROC curve was created using gene expression data from GSE17025

In total, 255 CTA genes were retrieved from the CTDatabase [29]. The transcriptional levels of these genes in EC tissues were compared to those in normal endometrial tissues using the TCGA database. In EC, 80 CTA genes were upregulated, whereas 17 genes were downregulated (Fig. 1A). We performed ssGSEA analysis using upregulated 80 CTA genes based on transcriptomic data from TCGA EC samples and grouped EC samples according to enrichment scores. Clinical association analysis showed that tumors with late-clinical stage and high grade, as well as mixed and serous tumors, tended to be significantly enriched in the group with high CTA score (CTA^high^) (Fig. 1B). In addition, the CTA^high^ group was substantially associated with shorter overall survival (especially in the first 10 years) in EC patients (Fig. 1C). Further differential gene analysis revealed that 45 of the 80 CTA genes were significantly highly expressed in the CTA^high^ group (Fig. 1D, E). To explore the main factors contributing to the considerably lower survival in the CTA^high^ group, we performed univariate cox regression on 45 upregulated genes and found three of them (*TTK*, *LY6K*, and *NOL4*) contributed to poor outcomes (Fig. 1F).

We obtained microarray data from the Gene Expression Omnibus (GEO) microarray dataset (GSE17025) cohort, which contains EC stage I and benign endometrial samples. *TTK* was shown to be significantly higher in stage I EC tissues compared to benign endometrial tissues, while the other two genes were not significantly differentially expressed in different tissues (Fig. 1G). Our survival analysis showed that high *TTK* expression was significantly associated with shorter overall survival in EC patients (Fig. 1H). In addition, *TTK* expression could also be used to distinguish early-stage EC tissues from normal tissues, with an AUC of 0.873 for early EC diagnosis (Fig. 1I). Our data showed that *TTK* is upregulated in EC and could be a potential biomarker for EC diagnosis and prognosis prediction. Therefore, *TTK* was chosen for further research.

### Aberrant *TTK* overexpression is linked to aggressive phenotypes in EC

**Fig. 2:**
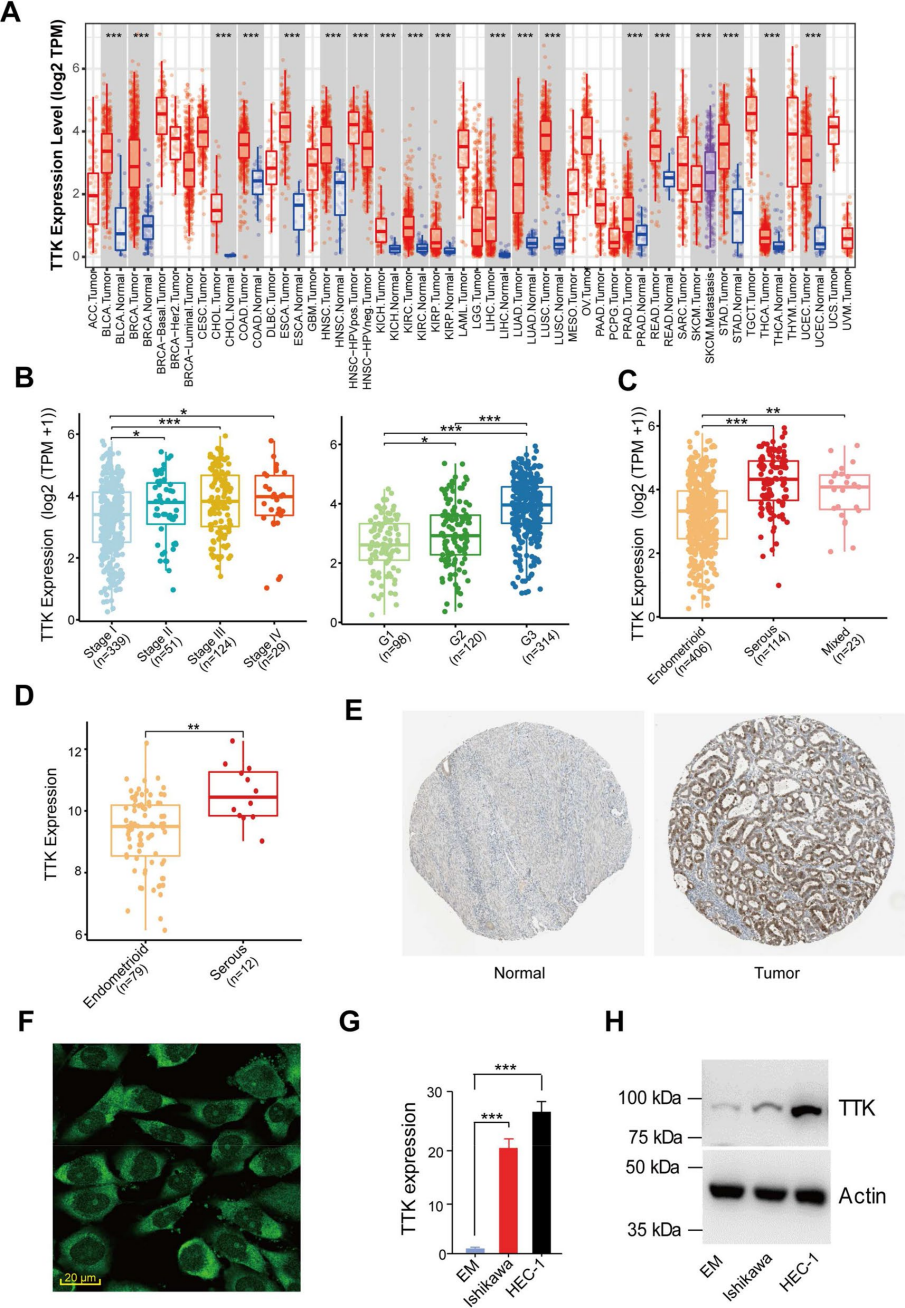
Aberrant *TTK* overexpression is linked to aggressive phenotypes in EC. **A** Differential analysis of *TTK* expression in TCGA pan-cancer. **B**, **C** Differential analysis of *TTK* expression in TCGA EC patients, as divided by tumor stage (**B**, left panel: stage I, stage II, stage III, and stage VI), tumor grade (**B**, right panel: G1, G2, and G3), as well as the histologic type (**C**; EEC: endometrioid; serous; mixed-type). **D** The GEO database was used to show increased *TTK* expression in serous ECs. The comparison was conducted using Wilcoxon tests. *P < 0.05, **P < 0.01, ***P < 0.001. **E** An immunohistochemical picture from the HPA database demonstrates high TTK protein expression in EC tissues than in adjacent normal tissues. **F** Results of subcellular localization of TTK protein, green indicates target protein, blue indicates nucleus and red indicates microtubule. Scale bars = 20 μm. **G**, **H** The mRNA (**G**) and protein (**H**) expression of TTK in two EC cell lines and a normal cell line EM were investigated using qRT-PCR and western blotting. ***P < 0.001, by Student’s *t-*tests

The TIMER1.0 database was used to examine the *TTK* mRNA expression levels in pan-cancers. The mRNA expression of *TTK* was significantly higher in almost all tumor types in TCGA compared to normal samples (Fig. 2A). *TTK* was expressed abundantly in EC samples with higher clinical stage and grade, but much less in ECs with lower clinical stage and grade (Fig. 2B). Serous EC, a rare but more aggressive subtype of EC, had a much higher level of *TTK* expression than endometrial endometrioid carcinomas (EECs) (Fig. 2C). In stage I EC samples (GSE17025), we validated that *TTK* levels were considerably increased in serous tumors than in EEC tumors (Fig. 2D). Using the Human Protein Atlas (HPA) database, we further investigated TTK protein expression in ECs. TTK levels in EC tissues were much higher than in adjacent normal tissues (Fig. 2E). In addition, the results of subcellular localization in the HPA database indicate that TTK was mainly localized in the nucleoli and cytosol (Fig. 2F). The qRT-PCR and western blotting experiments were then used to assess *TTK* expression levels in EM, a normal endometrial cell line, and two representative EC cell lines (Ishikawa and HEC-1). *TTK* mRNA and protein levels were significantly higher in the EC cell line compared to the EM cells (Fig. 2G, H). These results showed that high TTK expression is a promising biomarker for predicting aggressive EC.

### Involvement of TTK in the progression of the cell cycle in EC

**Fig. 3:**
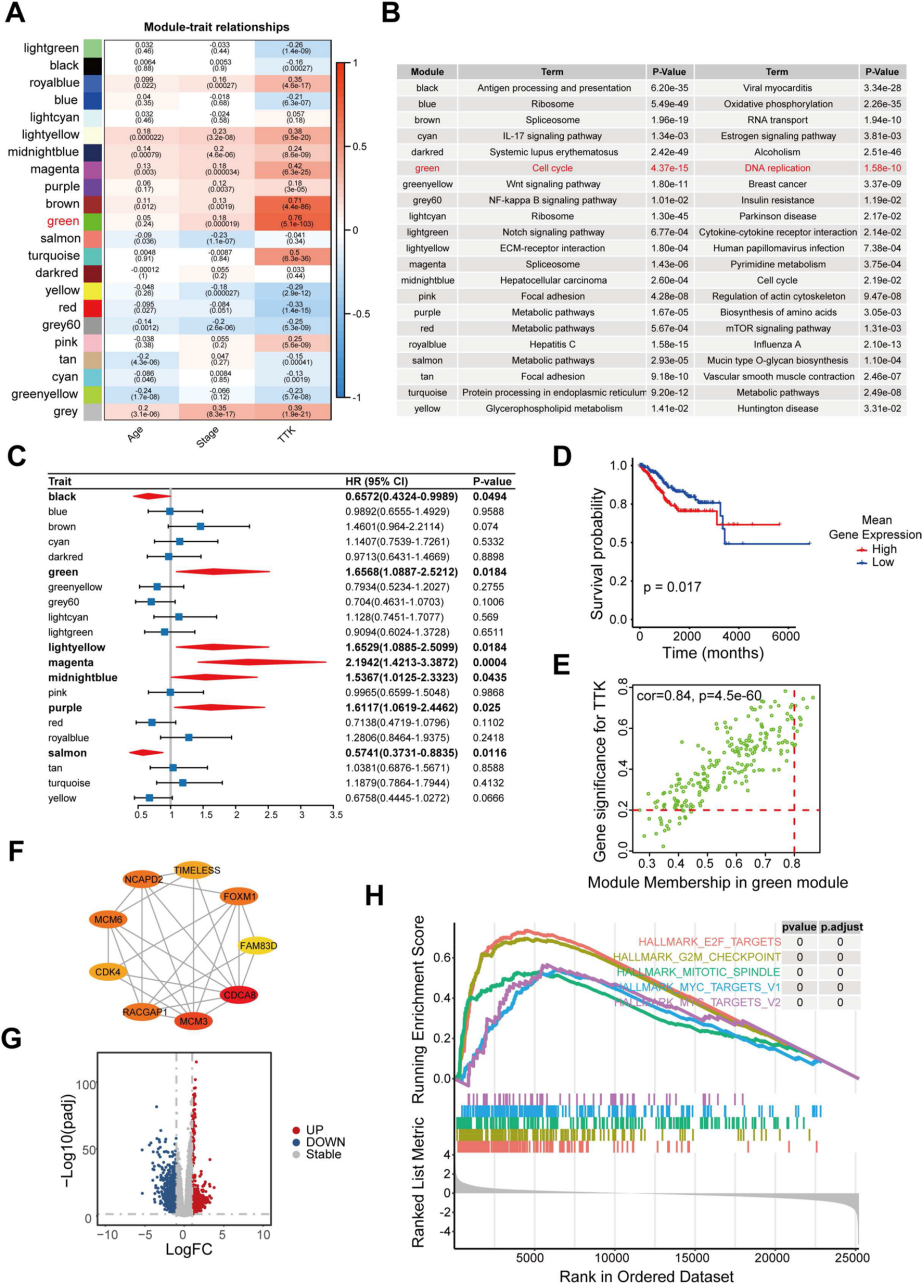
Involvement of TTK in the progression of the cell cycle in EC. **A** Heatmap shows the correlation of all modules and traits, with numbers in the modules indicating correlation coefficients and p-values. **B** KEGG enrichment analysis of genes in the modules revealed their biological functions. **C** Univariate cox regression analysis based on the mean expression of all genes in the modules. **D** Survival analysis of the green module gene set. A two-sided log-rank test is used to compare patient survival between the two groups. **E** The network plot shows the result of the protein–protein interaction analysis between the hub genes of the green module. **F** The scatter plot showed the correlation between GS values and MM of the green module; the horizontal red dashed line indicates GS = 0.2, and the vertical red dashed line indicates MM = 0.8. **G** Differential genes between the *TTK*^*high*^ group and TTK^low^ groups; red dots indicate genes significantly overexpressed in the *TTK*^*high*^ group, and blue dots indicate genes significantly overexpressed in the *TTK*^*low*^ group; the screening threshold is (log2|fold-change| > 1 and p-adjust < 0.05). The horizontal gray dashed line means *p*-adjust = 0.05, and the vertical gray dashed line means log2|fold-change| = 1. **H** Line chart illustrates the GSEA enrichment results; different colors indicate different hallmark terms

To investigate the biological function of TTK in EC, we performed co-expression network analysis (WGCNA) based on gene expression profiles of EC (Fig. 3A) and did KEGG enrichment for genes in each module (Fig. 3B). We found the most significant correlation between *TTK* and the green module (Pearson correlation = 0.76, *P* = 5.1e−103), which is mostly involved in cell cycle and DNA replication-related pathways (Fig. 3B). The results of univariate cox regression of the modules indicated that increased gene expression in the green module was strongly associated with shorter overall survival in EC patients (Fig. 3C), and this result was confirmed by the results of survival analysis (Fig. 3D). Meanwhile, we performed protein–protein interaction (PPI) network analysis on nine hub genes of the green module (Fig. 3E). The results of PPI analysis indicated that there were strong interactions among the hub genes in the green module (Fig. 3F).

By survival analysis, we found that high expression of three of the nine hub genes (*CDCA8*, *FAM83D*, and *RACGAP1*) was significantly associated with shorter overall survival (Additional file 1: Fig. S1A) and progression-free survival (Additional file 1: Fig. S1B) in EC patients. Similar to *TTK*, mRNA (Additional file 1: Fig. S1C) and protein (Additional file 1: Fig. S1D) expression of these three genes were significantly elevated in EC samples. Subcellular localization showed that CDCA8 was mainly localized in the nucleolus (Additional file 1: Fig. S1E). FAM83D was found in the mitotic spindle, and RACGAP1 was seen in the nucleoplasm. These results implicated the functions of these three proteins during mitosis.

In addition, we divided EC samples into TTK^high^ and TTK^low^ groups according to the gene expression level of *TTK* and performed differential expression analysis between the two groups (Fig. 3G). We found significant activation of cell proliferation-related pathways (including E2F Targets, G2M Checkpoint, Mitotic Spindle, MYC Targets V1/V2 pathways) in the TTK^high^ group according to GSEA enrichment analysis (Fig. 3H). TTK has been reported to maintain chromosome stability in mitosis and plays a critical role in chromosome alignment and spindle assembly checkpoints [30]. In addition, TTK could protect aneuploid cells from mitotic catastrophe, which leads to the development of aneuploid tumors [31]. These analyses imply that TTK facilitates the division of genomically unstable tumor cells to sustain the proliferation and progression of EC cells.

### Genomic and epigenetic alterations associated with *TTK* dysregulation in EC

**Fig. 4:**
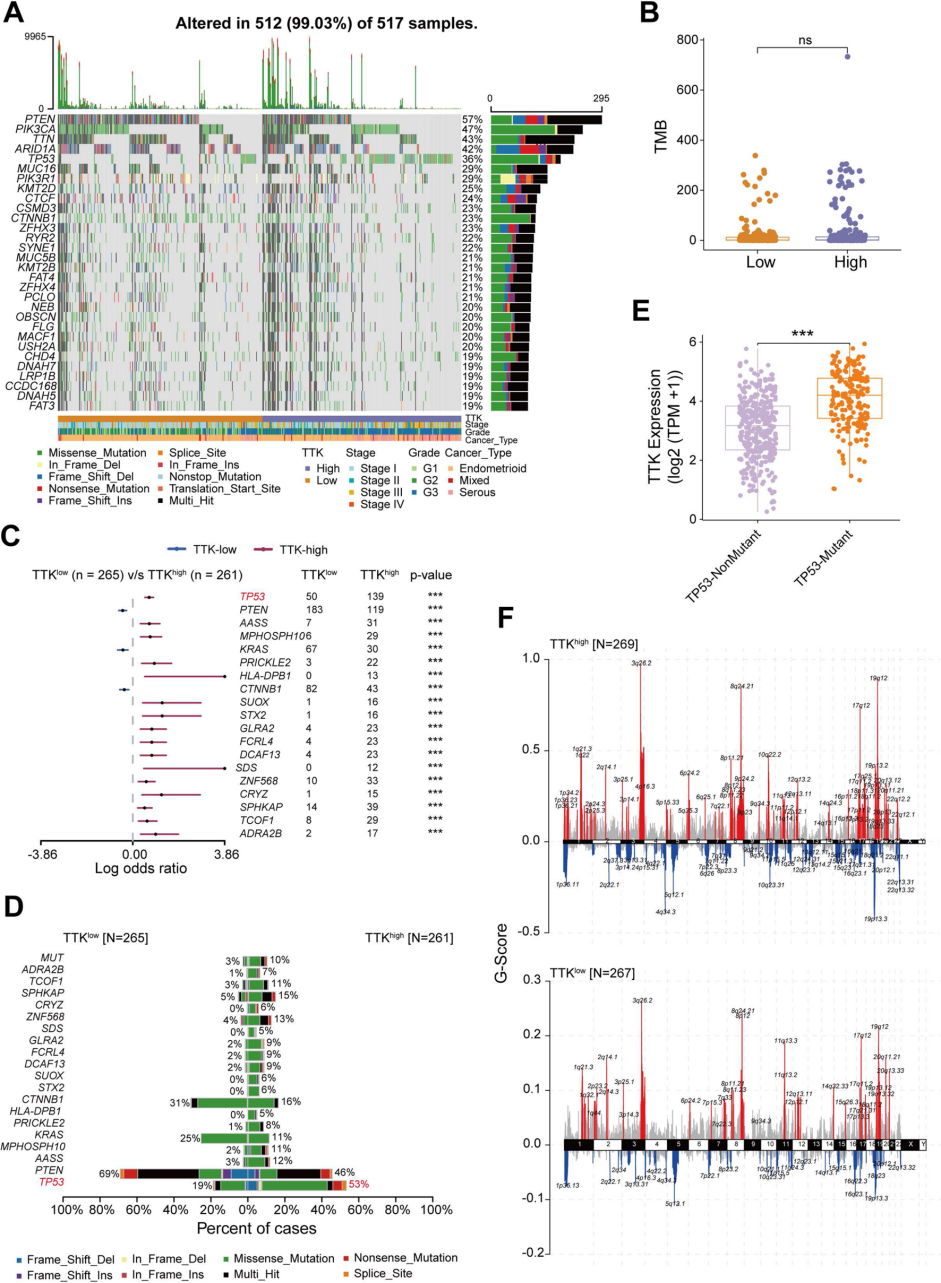
Genomic changes associated with dysregulated TTK expression in EC. **A** Top 30 SMG in TCGA EC. **B** The difference in TMB between the *TTK*^*high*^ and *TTK*^low^ groups. *ns* not significant. **C** Forest plot of the top 20 differentially mutated genes between *TTK*^high^ and *TTK*^low^ groups. **D** Cobar plot of the top 20 differentially mutated genes between *TTK*^high^ and *TTK*^low^ groups. **E** Differential analysis of *TTK* expression in TCGA EC patients as divided by TP53 mutation status (wild type and mutants). ***P < 0.001, by Wilcoxon tests. **F** The upper and lower chromosomes plot show the CNV landscape in samples from the *TTK*^*high*^ and *TTK*^low^ groups, respectively

To explore the genomic alterations related to TTK dysregulation in EC, we comprehensively analyzed the mutational landscape and compared between TTK^high^ and TTK^low^ groups based on somatic mutation data from TCGA EC samples (Fig. 4A). The results revealed no significant difference in tumor mutational burden (TMB) between the two groups of samples (Fig. 4B). In addition, we identified significantly mutated genes (SMGs) in the two groups of samples using MutsigCV (Additional file 1: Fig. S2A, B). The results of the fisher’s test suggested a significant difference in the mutation profiles of the two groups (Fig. 4C). The *TP53* mutation was significantly enriched in the TTK^high^ group, with a high mutation frequency of 53% (Fig. 4D). Further analysis revealed that TTK expression was significantly higher in the TP53 mutated group than others in ECs of TCGA (Fig. 4E). In addition, ECs from the TTK^high^ group had substantially higher tumor purity than that of the TTK^low^ group (Additional file 1: Fig. S2B). It’s suggested that dysregulation of TTK expression in ECs was related to TP53 mutations, which contributed to the persistent proliferation of tumor cells.

On the other hand, we compared the differences in copy number variations (CNVs) between the two groups based on CNV data (Fig. 4F) and compared the number of amplified and missing CNVs between the two groups of samples, respectively. We found that the number of both amplified and missing CNVs in the TTK^high^ group was significantly higher than that in the TTK^low^ group (Additional file 1: Fig. S2C). The comparison of CNVs showed that significant amplification occurred in the 1q22, 20q11, and 19q12 regions in the TTK^high^ group. In contrast, significant deletions occurred in the 16q21, 4q34, 17p13, 15q21, 9q34, and 9q21 regions in the TTK^low^ group (Additional file 1: Fig. S2D). We also found a significant difference between the population frequencies of CNV occurrence of cancer driver genes in the two sample groups (Additional file 1: Fig. S2E).

To further discover genomic alterations in the *TTK* gene across the pan-cancer population, we conducted gene mutation and copy number alteration analyses using the cBioPortal database. In 32 TCGA investigations, the *TTK* gene was altered in 306 (3%) of 10,953 individuals (Additional file 1: Fig. S3A). Almost all of the TCGA cancer patients had genetic alterations (“mutation and amplification”) (Additional file 1: Fig. S3A). Compared to patients with other cancer types, patients with EC had the greatest prevalence of *TTK* alterations (Additional file 1: Fig. S3A). Similar to established oncogenes (such as *TP53* and *MYC*), *TTK* missense mutation, amplification, and mRNA upregulation were seen in EC using Oncoprint data from cBioPortal (Additional file 1: Fig. S3B). Serous EC demonstrated greater rates of *TTK* gene amplification and *TTK* mRNA overexpression than EEC and mixed tumors (Additional file 1: Fig. S3C).

To explore epigenetic alterations associated with TTK dysregulation, we performed a genome-wide methylation analysis of all TCGA EC patients (Additional file 1: Fig. S4A) and found that 59 GpG sites located in the promoter region were differentially methylated (Additional file 1: Fig. S4B). Association analysis with differential genes between the two groups revealed that seven genes in which DMP are located were differentially expressed. Two of these genes (*ANG* and *C4BPB*) showed hypermethylation and down-regulation, and five genes (*BRINP1*, *KLHL1*, *NOL4*, *SLC6A15*, and *PKIA*) showed hypomethylation and up-regulation. Correlation analysis of methylation and gene expression suggested that gene expression of these seven genes was regulated by methylation of CpG sites located in the promoter region (Additional file 1: Fig. S4C, D). Survival analysis showed that low expression of two hypermethylated and down-regulated genes was associated with shorter overall survival in EC patients (Additional file 1: Fig. S4E). In comparison, the other five hypermethylated and upregulated genes were positively associated with worse survival (Additional file 1: Fig. S4F). These results suggest that the TTK^high^ and TTK^low^ groups show specific methylation profiles and methylation of the promoters of some genes could significantly affect gene expression and consequently affect the survival of patients.

### Cell subpopulations associated with dysregulated TTK expression in EC

**Fig. 5:**
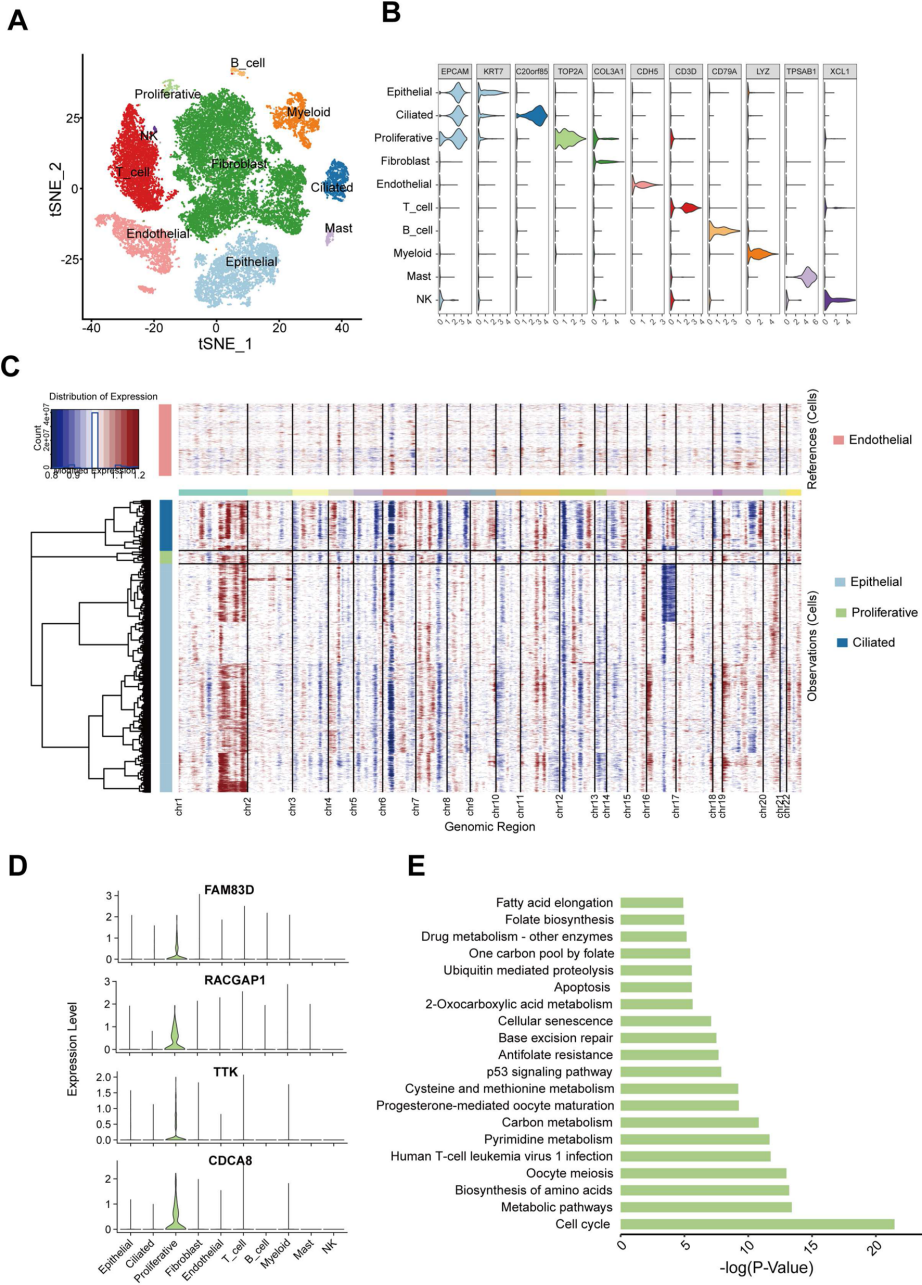
Cell subpopulations associated with TTK dysregulation in EC. **A** the t-SNE plot shows the cell types identified in EC with different colors. **B** The Violin plot illustrates the expression of classical marker genes in each cluster. **C** Heatmap showing large-scale CNV profiles of epithelial, ciliated, proliferative cells. Reference CNV levels of endothelial are presented at the top of the plot. The red color represents amplification and the blue represents deletion. **D** Violin plots depict *CDCA8*, *FAM83D*, *RACGAP1*, and *TTK* expression across major cell types. **E** The bar plot demonstrates the KEGG pathway enrichment of genes highly expressed in proliferative cells

Given that dysregulation of TTK expression is closely correlated with malignant proliferation of tumor cells and consequently increased tumor purity, we speculated that TTK might alter the proportion of cells in the tumor microenvironment. Based on this hypothesis, we analyzed single-cell transcriptome data of five EC samples from the GEO database. By using reported maker genes [32], we identified a total of 10 cell subpopulations (Fig. 5A, B), including epithelial cells, ciliated cells, proliferative cells, fibroblasts, endothelial cells, T cells, B cells, myeloid cells, mast cells, and NK cells.

Since EC is a malignant tumor of epithelial tissue origin, we used endothelial cells as the reference and inferred the CNV levels of epithelial, ciliated, and proliferative cells (Fig. 5C). The results showed that all three types of epithelial cells had amplification in the long arm of chromosome 1, which was consistent with the TCGA EC samples. However, we found different CNV in different types of epithelial cells. Ciliated cells had amplification in the short arm of chromosome 1 independently of the other two subpopulations, and parts of epithelial cells had a partial deletion of chromosome 16. In addition, it appears that a small proportion of normal diploid cells are present in the three types of epithelial cells. Moreover, we explored the expression of hub genes associated with *TTK* dysregulation across different cell types. Surprisingly, *CDCA8*, *FAM83D*, *RACGAP1*, and *TTK* were mainly expressed in proliferative cells (Fig. 5D). Functional enrichment analysis of genes highly expressed in proliferative cells showed that these genes are mainly associated with cell cycle and metabolic pathways (Fig. 5E). Hence, we speculate that this subpopulation may be related to tumor malignant proliferation and metastasis.

### High TTK expression is positively correlated with EMT signature and chemoresistance in EC

**Fig. 6:**
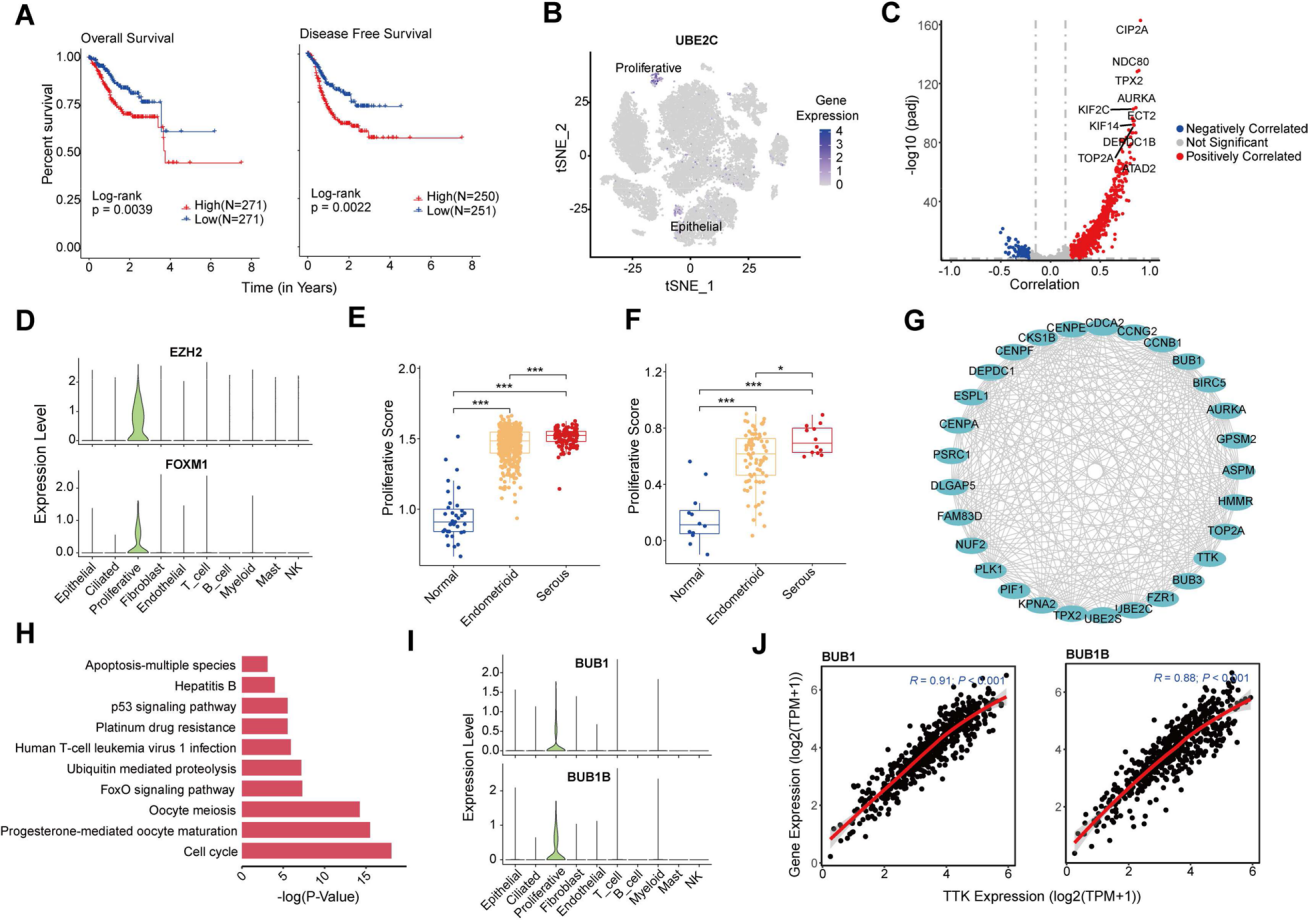
High TTK expression is positively correlated with EMT signature and chemoresistance in EC. **A** Analysis of patient survival based on *UBE2C* expression profile from the TCGA database. A two-sided log-rank test is used to compare patient survival between the two groups. **B** t-SNE plots show expression levels of *UBE2C* in major cell types. **C** Correlation analysis of *TTK* and EMT-related genes based on the gene expression profile of TCGA EC patients. Red dots indicate a significant positive correlation, while blue dots indicate the opposite. The screening threshold is (|correlation| > 0.2 and *p-*adjust < 0.05). The horizontal gray dashed line means *p-*adjust = 0.05, and the vertical gray dashed line means | correlation | = 0.2. The marked genes are the top 10 genes with the highest correlation. **D** Violin plots depict EZH2 and FOXM1 expression across major cell types. **E**, **F** Results of ssGSEA enrichment analysis of EC samples in TCGA database (**E**) and early stage of EC samples in GEO database (**F**) based on top 20 highly expressed genes of proliferative cell subpopulation. *P < 0.05, **P < 0.01, ***P < 0.001, by Wilcoxon tests. **G** Upregulated genes between cells with high *TTK* expression in the proliferative subpopulation relative to cells with low *TTK* expression. **H** Results of KEGG enrichment analysis of the upregulated genes in the E plot. **I** Violin plots depict *BUB1* and *BUBIB* expression across major cell types. **J** Correlation analysis of *TTK* and target genes (*BUB1* and *BUBIB*) in TCGA EC

To investigate the effect of different cell subpopulations on EC survival, we performed survival analysis of the top 5 highly expressed genes of all cell subpopulations based on the TCGA gene expression profile (Additional file 1: Fig. S5A). The results showed that only high expression of *UBE2C,* mainly expressed in proliferative cells, was substantially associated with shorter overall survival and progression-free survival of EC patients (Fig. 6A, B). It was reported that UBE2C promotes EMT via p53 in EC [33], suggesting that proliferative cells may have EMT phenotypes.

To verify the relationship between *TTK* gene expression and EMT in EC, we performed a correlation analysis of *TTK* and reported EMT-related genes [34] based on the gene expression profiles of TCGA EC data. We found that *TTK* showed a significant and positive correlation with the majority of EMT-related genes (Fig. 6C). We further investigated the expression of 19 genes with a correlation coefficient above 0.8 with TTK at the cellular level. Surprisingly, 18 of the 19 genes were significantly highly expressed in the proliferative cells (Additional file 1: Fig. S5B). In addition, the common EMT transcription factors EZH2 and FOXM1 were highly expressed in the proliferative subpopulation. We speculate that proliferative cells in EC might promote EMT by high expression of TTK. Therefore, we further performed ssGSEA analysis of EC and normal samples from the TCGA database based on the top 20 genes highly expressed in proliferating cell subpopulations. As expected, EC samples get a significantly higher proliferating score than normal tissues. At the same time, serous ECs are significantly higher than EEC tumors (Fig. 6E). And we also found a similar trend in the early EC samples (GSE17025) (Fig. 6F).

Moreover, we divided all proliferative cells into TTK^high^ and TTK^low^ groups according to their TTK expression levels, and 33 genes were significantly overexpressed in the TTK^high^ group, and strong protein–protein interaction exists among these genes (Fig. 6G). KEGG enrichment results suggested that these highly expressed genes may lead to platinum drug resistance (Fig. 6H). *BUB1* and *BUB1B* were highly expressed in proliferative cells (Fig. 6I), and they were shown to be associated with resistance to chemotherapeutic drugs, including TX, in previous studies [35–37]. Correlation analysis of gene expression profiles in TCGA EC samples also suggested that the expression of *TTK* showed a very significant positive correlation with *BUB1* and *BUB1B* (Fig. 6J), suggesting that high expression of *TTK* may lead to chemoresistance.

### Inhibition of TTK suppresses EMT and chemoresistance of EC cells

**Fig. 7:**
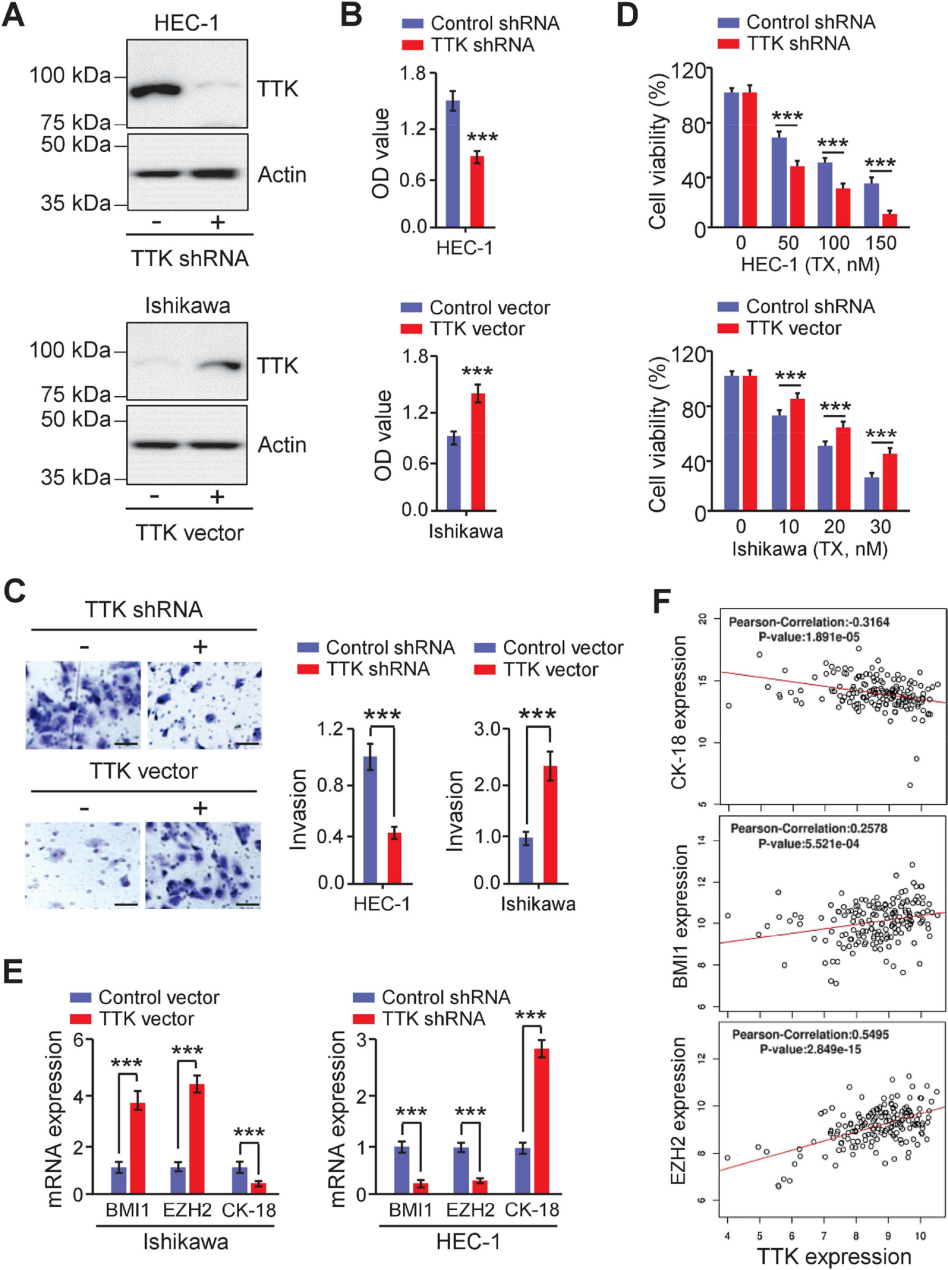
Inhibition of TTK suppresses EMT and chemoresistance of EC cells. **A** The protein expression of TTK was evaluated by western blotting in Ishikawa overexpressing TTK or HEC-1 cells transfected with TTK shRNA. **B**–**D** The CCK-8 (**B**), invasion (**C**), and MTT (**D**) assays were used to detect EC cell proliferation, invasion, and chemoresistance. Scale bars = 50 μm. ***P < 0.001, by Student’s *t-*tests. **E** qRT-PCR assays were used to investigate the expression of *CK-18*, *BMI-1*, and *EZH2* in Ishikawa transfected TTK overexpression vector (or control vector), and in HEC-1 cells transfected with TTK shRNA (or control shRNA). ***P < 0.001, by Student’s *t-*tests. **F** Correlation of *TTK* levels with *CK-18*, *BMI-1*, and *EZH2* expression in TCGA EC (LinkedOmics)

Previous research has shown that TTK has an important tumor-promoting role in triggering EMT in breast cancer [10] and bladder cancer [38]. As a result, we looked at how TTK inhibition affects EC cell proliferation, invasion, and chemoresistance. Two shRNAs were examined for their ability to lower TTK expression, and the one with the highest knockdown efficacy was selected for our study. TTK shRNA was then transfected into HEC-1 cells. Western blotting demonstrated that shRNA-mediated TTK knockdown reduced cell proliferation significantly in HEC-1 cells (Fig. 7A, B). Furthermore, inhibiting TTK expression significantly decreased the ability of HEC-1 cells to invade (Fig. 7C). TTK knockdown dramatically increased the sensitivity of HEC-1 cells to TX (Fig. 7D). The proliferation, invasion, and resistance to TX were consistently enhanced in Ishikawa cells transfected with the TTK-overexpressing vector (Fig. 7A–D). Changes in several EMT-related genes in TTK-overexpressing Ishikawa cells and HEC-1 cells with TTK knockdown were examined to see whether TTK influences the EMT process. In HEC-1 cells with TTK knockdown, qRT-PCR assays demonstrated an increase in epithelial marker *CK-18* and a decrease in recognized EMT inducers (*BMI-1* and *EZH2*) (Fig. 7E). The opposite gene expression patterns were seen in TTK-overexpression Ishikawa cells (Fig. 7E). We next used the LinkedOmics database to assess whether *TTK* expression was associated with *CK-18*, *BMI-1*, and *EZH2* levels in TCGA EC tissues. In EC samples, we found strong positive associations between *TTK* and *BMI-1* (or *EZH2*) expression (Fig. 7F). *TTK* expression was shown to be negatively associated with *CK-18* expression in EC (Fig. 7F).

To further test the tumor-promoting role of TTK, we conducted a series of experiments to test whether inhibiting TTK activity with its specific inhibitor AZ3146 might replicate the effects of TTK downregulation. The cytotoxicity of the TTK inhibitor AZ3146 was assessed with MTT assays. After 24 h of treatment with different concentrations of AZ3146 on EC cells, we determined that doses of 30 nM (for HEC-1 cells) or 15 nM (for Ishikawa cells) were cytotoxic (Additional file 1: Fig. S6A). In later experiments, a subtoxic dose of AZ3146 (28 nM for HEC-1 cells and 14 nM for Ishikawa cells) was used to test its effects on EC cells. CCK-8 experiments revealed that AZ3146 efficiently inhibited EC cell growth (Additional file 1: Fig. S6B). Subsequent cell invasion studies demonstrated that AZ3146 administration dramatically inhibited cell invasiveness (Additional file 1: Fig. S6C). To determine the influence of AZ3146 on the apoptosis of EC cells, apoptotic assays were used. As expected, the activity of Caspase-3/7 in EC cells was increased upon TX treatment (Additional file 1: Fig. S6D). Interestingly, when EC cells were treated with both TX and AZ3146, Caspase-3/7 activity was further enhanced, suggesting that AZ3146 has a synergistic impact on TX-induced cell apoptosis (Additional file 1: Fig. S6D). All these data suggested that TTK is a unique EMT activator in EC and that inhibition of TTK diminishes the invasive, mesenchymal and chemoresistant properties of EC cells.

### TTK induces EMT in EC cells by upregulating miR-21 expression

**Fig. 8:**
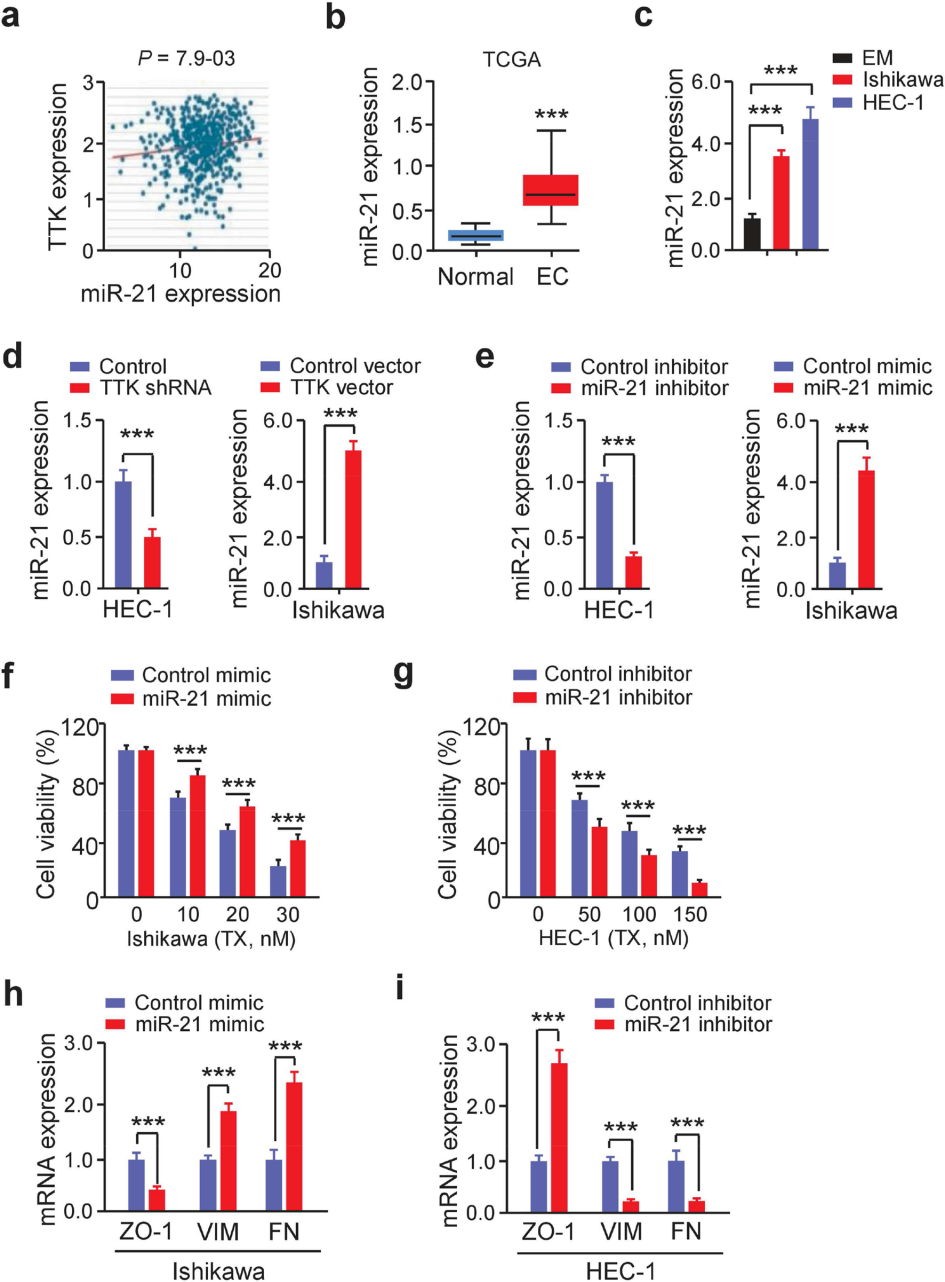
TTK induces EMT in EC cells by upregulating miR-21 expression. **A** Correlation of *TTK* levels with miR-21 expression in TCGA EC (ENCORI). **B** The expression of miR-21 in TCGA EC and normal samples (UALCAN). ***P < 0.001, by Wilcoxon tests. **C** MiR-21 levels in EC cells and EM cells as assessed by qRT-PCR. ***P < 0.001, by Student’s *t-*tests. **D** The expression of miR-21 in EC cells transfected with TTK expression vector (or control vector), or with TTK shRNA (control shRNA). ***P < 0.001, by Student’s *t-*tests. **E** MiR-21 expression was evaluated using qRT-PCR in EC cells transfected with miR-21 mimic (or miR-21 inhibitor). ***P < 0.001, by Student’s *t-*tests. **F**, **G** MTT assays were conducted to examine the effects of miR-21 overexpression (**F**) or knockdown (**G**) on TX resistance. ***P < 0.001, by Student’s *t-*tests. **H**, **I** qRT-PCR was performed to measure the expression of *ZO-1*, *VIM*, and *FN* in EC cells transfected with miR-21 mimic (**H**) or with miR-21 inhibitor (**I**). ***P < 0.001, by Student’s *t-*tests

TTK induces EMT in breast cancer cells by upregulating miR-21 and decreasing miR-200c levels [10]. Thus, we examined the ENCORI database to determine whether *TTK* and miR-21 and miR-200c expression in EC samples were clinically relevant. There was a significant positive relationship between *TTK* mRNA expression and miR-21 (but not miR-200c) expression in 538 EC samples (Fig. 8A). MiR-21 expression was significantly higher in EC tissues than in normal tissues (Fig. 8B). Using qRT-PCR, we also found that miR-21 was expressed at greater levels in EC cells than in a normal cell line EM (Fig. 8C). To investigate whether TTK inhibition affects EMT through miR-21, qRT-PCRs were used to quantify miR-21 levels in EC cells overexpressing TTK or transfected with TTK shRNA. TTK overexpression was shown to increase the expression of miR-21 in Ishikawa cells (Fig. 8D). TTK shRNA transfection resulted in a substantial reduction in miR-21 expression in HEC-1 cells (Fig. 8D). MiR-21 has previously been demonstrated to be overexpressed in EC samples when compared to non-tumor tissues [39], and high miR-21 expression in EC tissues has been associated to poor survival [40]. Furthermore, it has been shown that miR-21 is an EMT activator and that blocking miR-21 in EC cells reverses EMT [43]. To find out whether miR-21 has a role in chemoresistance, EC cells were transfected with either an inhibitor or a mimic of miR-21. The qRT-PCRs validated that miR-21 was downregulated or upregulated in the corresponding EC cells (Fig. 8E). Using a cell viability experiment, we found that miR-21 overexpression reduced the sensitivity to TX in Ishikawa cells, whereas miR-21 deletion in HEC-1 cells eliminated TX resistance (Fig. 8F, G). In Ishikawa cells with miR-21 overexpression, our qRT-PCR tests revealed a reduction in *ZO-1* expression as well as an increase in *VIM* and *FN* abundance (Fig. 8H). In contrast, suppressing miR-21 in HEC-1 cells dramatically increased *ZO-1* expression while decreasing *VIM* and *FN* expression (Fig. 8I). Together, our findings showed that increased miR-21 expression is required for TTK to initiate EMT and chemoresistance.

## Discussion

**Fig. 9:**
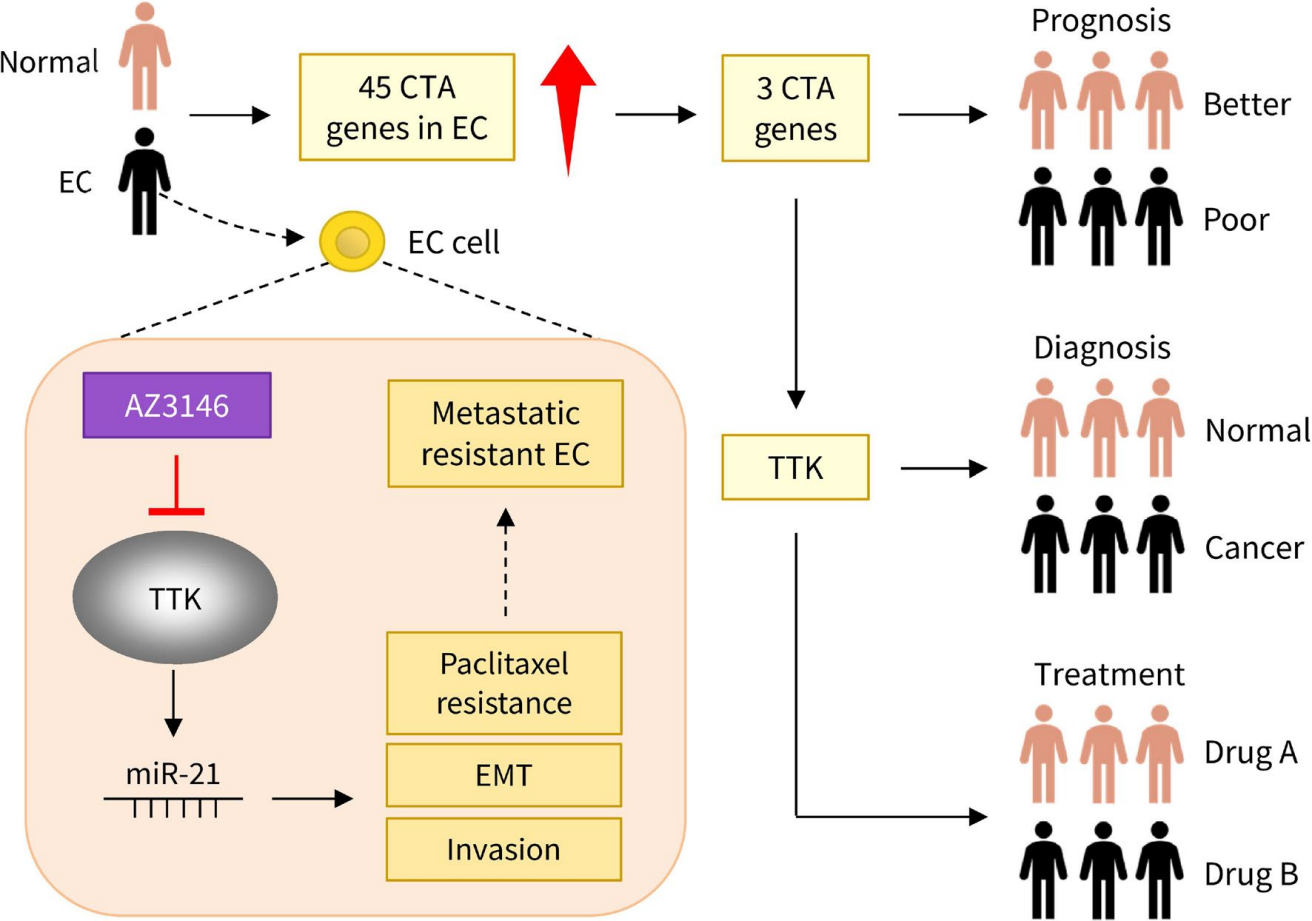
Illustration demonstrating the role of the TTK/miR-21 pathway in facilitating EMT and conferring chemoresistance in EC. Through a screening process, TTK was discovered to be a potential biomarker and therapeutic target for EC. Higher TTK expression in EC cells leads to increased miR-21 expression, which induces EMT, cell proliferation, and chemoresistance

ECs represent very serious health challenges, and new biomarkers are required to allow for early detection, as well as new approaches to improve the sensitivity of chemotherapeutic agents, which could significantly enhance the opportunities for successful cancer treatment and favor a higher chance of cure, survival, and quality of life for patients [3]. Increasing evidence suggests that CTAs play a crucial role in tumorigenesis and progression. Many CTAs are known to promote the progression of tumors through cellular activities such as cell proliferation, division, or cell motility [6, 7]. CTA gene dysregulation is a common occurrence in cancer and significantly decreases the survival of patients [6]. However, the functions of CTA genes and their potential therapeutic uses in EC are currently unclear. In this study, we found that high expression of dysregulated CTA (including *TTK*) genes in EC was significantly associated with the advanced stage of EC and shorter survival. Moreover, our findings hinted at the tumor-promoting functions of TTK in EC (Fig. 9).

Numerous prior research has examined the association between TTK expression and cancer diagnosis [41, 42]. Earlier research demonstrated the diagnostic usefulness of TTK in differentiating liver cancer tissues from normal liver tissues [41]. Increased TTK expression has high diagnostic efficacy for breast cancer screening [42]. However, the potential diagnostic usefulness of the TTK in EC has not been investigated. In our study, we established that TTK had accurate diagnostic utility for stage I EC, thus indicating that highly expressed TTK can be used as a valuable biomarker for early diagnosis of EC.

Previous studies involving TTK have not only examined its value for cancer diagnosis, but also analyzed its role in determining the prognosis of cancer patients. A higher histological stage, a greater number of metastatic lymph nodes, and a shorter 5-year overall survival in lung cancer are all linked to high TTK expression [43]. In addition, overexpression of TTK is connected to cancer progression and poor prognosis in triple-positive breast cancer patients [44]. In ovarian cancer tissues and cells, TTK expression was upregulated, and its overexpression was discovered to be related to an unfavorable prognosis in ovarian cancer patients [45]. Based on our findings, patients with EC who have high TTK expression have a worse probability of surviving. Consequently, our study emphasized the potential role of TTK in assessing EC outcomes (Fig. 9).

TTK plays a significant role in EMT as well as the processes of metastasis and chemoresistance [10, 11]. TTK expression has been reported to be strongly elevated in EC tissues and high TTK expression was associated with significantly poor prognosis [12]. In addition, an interesting paper yielded similar results as we did. A recent study discovered that inhibition of TTK expression significantly suppressed the proliferation of EC cells [13]. Similar to these previous studies, our findings suggested that TTK plays a critical role in EC cell proliferation, EMT, and TX resistance. Overall, we demonstrated that inhibiting TTK may prevent EC metastasis and chemotherapy (Fig. 9).

We investigated the link between dysregulated CTA gene levels and EC patient prognosis. According to our data, dysregulated CTAs scores in EC correlated with higher tumor stage and lower overall survival. *TTK* was also discovered to be highly expressed in the CTA^high^ group, and the expression of this gene was significantly associated with lower survival. In addition, despite the great progress in understanding molecular tumor processes throughout the years, there are still no biomarkers routinely used to detect EC early. In this study, mRNA levels of *TTK* were greatly increased in early-stage EC compared to the benign tissues. Furthermore, our ROC analysis revealed that *TTK* was extremely accurate in distinguishing stage I EC tissues from benign endometrial samples, suggesting that TTK might be a biomarker for the early detection of EC.

Through WGCNA analysis and GSEA enrichment analysis of transcriptome data from EC samples in TCGA, we found that TTK in EC samples mainly regulates the cell cycle by regulating mitotic checkpoints, thus promoting tumor progression. In addition, we explored the reasons for the shortened survival of EC caused by TTK dysregulation in EC samples at the mutational, CNV, and epigenetic levels, respectively. Our analysis confirmed that higher TTK expression was more frequently detected in the poorly differentiated tumors, advanced stage of disease, *TP53*-mutated, and serous ECs. Thus, this study also suggests that TTK might be employed to predict EC development and metastasis.

Recent applications of single-cell transcriptome analysis in gynecological cancers include elucidating the heterogeneity of the disease, collecting microenvironmental data, elucidating drug resistance mechanisms, and enhancing cancer diagnostic and treatment approaches [46]. Previous research found that EC cells could be classified into several cell types: epithelial cells, fibroblasts, immune cells, and endothelial cells [47]. However, an in-depth analysis of epithelial EC cells at the single-cell level is currently lacking. Here, by studying the transcriptomes of single cells, we discovered the intra-tumor heterogeneity of EC and demonstrated that EC is comprised of different cell types (epithelial cells, immune cells, fibroblasts, and endothelial cells). In addition, we uncover a rare cluster of EC cells with proliferative signatures. Interestingly, we showed that *TTK*, hub genes, and EMT-associated genes were enriched in this proliferative subpopulation, implying that these cells tend to have more aggressive behavior. Our transcriptomic analysis also revealed that genes highly expressed in proliferative cells are more often expressed by serous ECs, rather than EEC tumors. In line with our findings, Olbrecht et al. recognized a molecular subtype of ovarian cancers based on single-cell RNA sequencing data and they reported that tumor sub-clusters expressing proliferative genes were enriched for EMT, hypoxia, and hedgehog signaling, all of which contribute to the greater aggressiveness of ovarian cancer [48]. These results support that a subpopulation of EC cells with proliferative capability may be responsible for accelerated metastatic development and TTK overexpression may be a valuable biomarker for predicting a highly aggressive nature.

A phenomenon called partial EMT or hybrid EMT, in which epithelial cancer cells display both mesenchymal and epithelial features, has been reported [49, 50]. Previous research has shown that partial EMT is more common in ECs than complete EMT [51]. Based on single-cell data from EC samples in the GEO database, we identified a subpopulation of proliferating malignant epithelial cells in EC that increases with tumor malignancy. The upregulation of *TTK* and numerous EMT-related genes was detected in proliferative cells, indicating that this subpopulation retains high proliferative potentials, while undergoing an EMT program. When proliferative cells were categorized according to their *TTK* expression levels, we discovered that the overexpressed genes in the TTK^high^ group were functionally engaged in regulating chemoresistance. These results collectively supported the notion that activation of TTK and its downstream pathways might contribute to the proliferation, EMT features, and chemoresistance of EC cells. In line with this possibility, cell functional experiments have proven that silencing TTK with shRNA significantly attenuated the proliferative and invasive properties and abolished the resistance to the anti-cancer drug TX. Animal models would be required in the future to demonstrate whether TTK silencing or specific inhibition may prevent EC metastasis and boost chemotherapeutic effectiveness.

Typically, TTK and other cancer-testis antigens are expressed in testicular and tumor but not in normal [7–11]. TTK has been implicated in the activation of EMT in breast [10] and bladder cancers [38]. In the current investigation, we found that TTK promotes EMT in EC cells by increasing the expression of EMT inducers (*EZH2* and *BMI-1*) and decreasing the expression of the epithelial marker *CK-18*. These results imply that reducing TTK expression could potentially be developed as a new strategy to specifically inhibit the metastasis of EC. The mechanism by which TTK enhances EC aggressiveness and chemoresistance through EZH2 overexpression remains to be determined.

Prior studies have shown that ECs with high levels of miR-21 expression had advanced tumor stages, high histological grades, cervical invasion, myometrial invasion, and distant metastasis [52]. As a result, miR-21 might be used as a biomarker to identify benign lesions from ECs [52]. MiR-21 has been shown to promote the growth of esophageal carcinoma [53], melanoma [54], and cervical cancer [55]. MiR-21 promotes EMT in renal cell cancer [56]. MiR-21 confers doxorubicin resistance in gastric cancer [57]. In accordance with these results, we demonstrated that miR-21 overexpression was responsible for EMT development and TX resistance, suggesting that miR-21 inhibitors may be useful in increasing chemotherapeutic effectiveness in EC patients.

The mechanisms behind miR-21 overexpression in tumor cells have been reported [58, 59]. The oncogene *RAS* has been associated with miR-21 overexpression in a thyroid cell line generated from the thyroid gland [58]. In addition, EZH2 increases miR-21 levels in glioma cells by epigenetically inhibiting the expression of a long non-coding RNA called MEG3 [59]. MEG3 decreased miR-21 levels in chronic myeloid leukemia cells [60]. Our findings showed that TTK promotes EZH2 expression and causes miR-21 to be upregulated. Thus, TTK may activate the miR-21-dependent signaling pathway through EZH2. Notably, miR-21 has been shown to increase EZH2 expression in human lung cancer stem cells [61]. Therefore, when EZH2 and miR-21 work together, they may create a double-positive feedback loop that mediates the oncogenic activities of TTK and enhances EC aggressiveness and chemoresistance. However, further experimental verification is needed.

Numerous small-molecule drugs that suppress TTK activity have been discovered or created so far [62–65]. The selective TTK inhibitor CFI-402257 has anti-cancer effects on human colon cancer cells, causing cell death [62]. A TTK-targeting small drug (compound 13) dramatically suppressed breast cancer development in nude mice [63]. According to some research, combining TTK inhibitors with chemotherapeutic drugs may significantly boost the effectiveness of destroying tumor cells [64]. BAY-1217389 and CFI-402257, two selective TTK inhibitors, greatly decreased glioblastoma multiforme cell proliferation and enhanced the growth-suppressive efficacy of temozolomide [64]. Moreover, Mps1-IN-3, a selective small molecule TTK inhibitor, produced mitotic aberrations in glioblastoma cells, and its combination with vincristine increased cell death [65]. Apart from these reports, Multiple breast cancer cell lines with a basal-like phenotype were more radiosensitive after TTK inhibition by B909 [66]. In line with recent findings from EC, which demonstrated that a TTK inhibitor (NTRC0066-0) significantly reduced the growth of EC cells [13], we confirmed that pharmacological inhibition of TTK with AZ3146 decreased the proliferation and invasion of EC in vitro, and had a synergistic effect on the TX-induced apoptosis of EC cells. Therefore, TTK inhibitors could be used with existing anti-tumor drugs or radiation therapy to enhance tumor destruction. The future use of AZ3146 in conjunction with TX may improve the therapeutic benefits of TX in EC patients exhibiting high TTK expression (Fig. 9).

## Conclusions

In summary, TTK was discovered as a new EMT activator for EC based on integrative multi-omics analysis and functional validation. TTK has the potential to be a valuable biomarker for the diagnosis of EC, the assessment of prognosis, and the prediction of chemoresistance (Fig. 9). Therefore, this study deepens our understanding of the biological function of TTK in EC and may provide new clues for treating this disease.

## Supplementary Information

**Additional file 1: Figure S1.** Expression of specific genes in EC. **Figure S2.** Differences in SMG and CNV associated with dysregulated TTK gene expression in EC. **Figure S3.** Extensive genomic changes of *TTK* gene in human cancers. **Figure S4.** Epigenetic differences associated with TTK dysregulation in EC. **Figure S5.** EMT-related genes expression across major cell types. **Figure S6.** Pharmacologic inhibition of TTK mimicked the effects of TTK silencing on EC cells.

## Data Availability

The datasets used and/or analyzed during the current study are available from the corresponding author on reasonable request. The public datasets analyzed during the current study are available in the repositories listed below: Gene Expression Omnibus: https://www.ncbi.nlm.nih.gov/geo/. The Cancer Genome Atlas: http://cancergenome.nih.gov/.

## Abbreviations

EMT: Epithelial-to-mesenchymal transition
EC: Endometrial cancer
EEC: Endometrial endometrioid carcinomas
TCGA: The Cancer Genome Atlas
GEO: Gene Expression Omnibus

## References

[1] Sung H, Ferlay J, Siegel RL, Laversanne M, Soerjomataram I, Jemal A, Bray F (2021). Global cancer statistics 2020: GLOBOCAN estimates of incidence and mortality worldwide for 36 cancers in 185 countries. CA Cancer J Clin.

[2] Rodriguez-Freixinos V, Karakasis K, Oza AM (2016). New targeted agents in endometrial cancer: are we really making progress?. Curr Oncol Rep.

[3] Gadducci A, Cosio S (2021). Pharmacological treatment of advanced, persistent or metastatic endometrial cancer state of the art and perspectives of clinical research for the special issue “diagnosis and management of endometrial cancer”. Cancers.

[4] Dong P, Xiong Y, Konno Y, Ihira K, Kobayashi N, Yue J, Watari H (2021). Long non-coding RNA DLEU2 drives EMT and glycolysis in endometrial cancer through HK2 by competitively binding with miR-455 and by modulating the EZH2/miR-181a pathway. J Exp Clin Cancer Res.

[5] Dong P, Kaneuchi M, Watari H, Hamada J, Sudo S, Ju J, Sakuragi N (2011). MicroRNA-194 inhibits epithelial to mesenchymal transition of endometrial cancer cells by targeting oncogene BMI-1. Mol Cancer.

[6] Salmaninejad A, Zamani MR, Pourvahedi M, Golchehre Z, Hosseini Bereshneh A, Rezaei N (2016). Cancer/testis antigens: expression, regulation, tumor invasion, and use in immunotherapy of cancers. Immunol Invest.

[7] Fan C, Qu H, Wang X, Sobhani N, Wang L, Liu S, Xiong W, Zeng Z, Li Y (2021). Cancer/testis antigens: from serology to mRNA cancer vaccine. Semin Cancer Biol.

[8] Iwata T, Fujita T, Hirao N, Matsuzaki Y, Okada T, Mochimaru H, Susumu N, Matsumoto E, Sugano K, Yamashita N, Nozawa S, Kawakami Y (2005). Frequent immune responses to a cancer/testis antigen, CAGE, in patients with microsatellite instability-positive endometrial cancer. Clin Cancer Res.

[9] Li S, Meng L, Zhu C, Wu L, Bai X, Wei J, Lu Y, Zhou J, Ma D (2010). The universal overexpression of a cancer testis antigen hiwi is associated with cancer angiogenesis. Oncol Rep.

[10] King JL, Zhang B, Li Y, Li KP, Ni JJ, Saavedra HI, Dong JT (2018). TTK promotes mesenchymal signaling via multiple mechanisms in triple negative breast cancer. Oncogenesis.

[11] Liu Y, Zhu K, Guan X, Xie S, Wang Y, Tong Y, Guo L, Zheng H, Lu R (2021). TTK is a potential therapeutic target for cisplatin-resistant ovarian cancer. J Ovarian Res.

[12] Yang Q, Yu B, Sun J (2020). TTK, CDC25A, and ESPL1 as prognostic biomarkers for endometrial cancer. Biomed Res Int.

[13] Cui CH, Wu Q, Zhou HM, He H, Wang Y, Tang Z, Zhang Y, Wang X, Xiao J, Zhang H (2022). High tyrosine threonine kinase expression predicts a poor prognosis: a potential therapeutic target for endometrial carcinoma. Ann Transl Med.

[14] Murali R, Delair DF, Bean SM, Abu-Rustum NR, Soslow RA (2018). Evolving roles of histologic evaluation and molecular/genomic profiling in the management of endometrial cancer. J Natl Compr Cancer Netw.

[15] Love MI, Huber W, Anders S (2014). Moderated estimation of fold change and dispersion for RNA-seq data with DESeq2. Genome Biol.

[16] Langfelder P, Horvath S (2008). WGCNA: an R package for weighted correlation network analysis. BMC Bioinform.

[17] Robin X, Turck N, Hainard A, Tiberti N, Lisacek F, Sanchez JC, Müller M (2011). pROC: an open-source package for R and S+ to analyze and compare ROC curves. BMC Bioinform.

[18] Lawrence MS, Stojanov P, Polak P, Kryukov GV, Cibulskis K, Sivachenko A, Carter SL, Stewart C, Mermel CH, Roberts SA, Kiezun A, Hammerman PS, McKenna A, Drier Y, Zou L, Ramos AH, Pugh TJ, Stransky N, Helman E, Kim J, Sougnez C, Ambrogio L, Nickerson E, Shefler E, Cortés ML, Auclair D, Saksena G, Voet D, Noble M, DiCara D, Lin P, Lichtenstein L, Heiman DI, Fennell T, Imielinski M, Hernandez B, Hodis E, Baca S, Dulak AM, Lohr J, Landau DA, Wu CJ, Melendez-Zajgla J, Hidalgo-Miranda A, Koren A, McCarroll SA, Mora J, Crompton B, Onofrio R, Parkin M, Winckler W, Ardlie K, Gabriel SB, Roberts CWM, Biegel JA, Stegmaier K, Bass AJ, Garraway LA, Meyerson M, Golub TR, Gordenin DA, Sunyaev S, Lander ES, Getz G (2013). Mutational heterogeneity in cancer and the search for new cancer-associated genes. Nature.

[19] Mayakonda A, Lin DC, Assenov Y, Plass C, Koeffler HP (2018). Maftools: efficient and comprehensive analysis of somatic variants in cancer. Genome Res.

[20] Skidmore ZL, Wagner AH, Lesurf R, Campbell KM, Kunisaki J, Griffith OL, Griffith M (2016). GenVisR: genomic visualizations in R. Bioinformatics.

[21] Mermel CH, Schumacher SE, Hill B, Meyerson ML, Beroukhim R, Getz G (2011). GISTIC2.0 facilitates sensitive and confident localization of the targets of focal somatic copy-number alteration in human cancers. Genome Biol.

[22] Tian Y, Morris TJ, Webster AP, Yang Z, Beck S, Feber A, Teschendorff AE (2017). ChAMP: updated methylation analysis pipeline for Illumina BeadChips. Bioinformatics.

[23] Yu G, Wang LG, Han Y, He QY (2012). clusterProfiler: an R package for comparing biological themes among gene clusters. OMICS.

[24] Hänzelmann S, Castelo R, Guinney J (2013). GSVA: gene set variation analysis for microarray and RNA-seq data. BMC Bioinform.

[25] McGinnis CS, Murrow LM, Gartner ZJ (2019). DoubletFinder: doublet detection in single-cell RNA sequencing data using artificial nearest neighbors. Cell Syst.

[26] Hao Y, Hao S, Andersen-Nissen E, Mauck WM, Zheng S, Butler A, Lee MJ, Wilk AJ, Darby C, Zager M, Hoffman P, Stoeckius M, Papalexi E, Mimitou EP, Jain J, Srivastava A, Stuart T, Fleming LM, Yeung B, Rogers AJ, McElrath JM, Blish CA, Gottardo R, Smibert P, Satija R (2021). Integrated analysis of multimodal single-cell data. Cell.

[27] Korsunsky I, Millard N, Fan J, Slowikowski K, Zhang F, Wei K, Baglaenko Y, Brenner M, Loh PR, Raychaudhuri S (2019). Fast, sensitive and accurate integration of single-cell data with Harmony. Nat Methods.

[28] Patel AP, Tirosh I, Trombetta JJ, Shalek AK, Gillespie SM, Wakimoto H, Cahill DP, Nahed BV, Curry WT, Martuza RL, Louis DN, Rozenblatt-Rosen O, Suvà ML, Regev A, Bernstein BE (2014). Single-cell RNA-seq highlights intratumoral heterogeneity in primary glioblastoma. Science.

[29] Almeida LG, Sakabe NJ, deOliveira AR, Silva MC, Mundstein AS, Cohen T, Chen YT, Chua R, Gurung S, Gnjatic S, Jungbluth AA, Caballero OL, Bairoch A, Kiesler E, White SL, Simpson AJ, Old LJ, Camargo AA, Vasconcelos AT (2009). CTdatabase: a knowledge-base of high-throughput and curated data on cancer-testis antigens. Nucleic Acids Res.

[30] Anderhub SJ, Mak GW, Gurden MD, Faisal A, Drosopoulos K, Walsh K, Woodward HL, Innocenti P, Westwood IM, Naud S, Hayes A, Theofani E, Filosto S, Saville H, Burke R, van Montfort RLM, Raynaud FI, Blagg J, Hoelder S, Eccles SA, Linardopoulos S (2019). High proliferation rate and a compromised spindle assembly checkpoint confers sensitivity to the MPS1 inhibitor BOS172722 in triple-negative breast cancers. Mol Cancer Ther.

[31] Janssen A, Kops GJ, Medema RH (2009). Elevating the frequency of chromosome mis-segregation as a strategy to kill tumor cells. Proc Natl Acad Sci USA.

[32] Fitzgerald HC, Dhakal P, Behura SK, Schust DJ, Spencer TE (2019). Self-renewing endometrial epithelial organoids of the human uterus. Proc Natl Acad Sci USA.

[33] Liu Y, Zhao R, Chi S, Zhang W, Xiao C, Zhou X, Zhao Y, Wang H (2020). UBE2C Is upregulated by estrogen and promotes epithelial–mesenchymal transition via p53 in endometrial cancer. Mol Cancer Res.

[34] Zheng G, Ma Y, Zou Y, Yin A, Li W, Dong D (2018). HCMDB: the human cancer metastasis database. Nucleic Acids Res.

[35] Bargiela-Iparraguirre J, Prado-Marchal L, Pajuelo-Lozano N, Jiménez B, Perona R, Sánchez-Pérez I (2014). Mad2 and BubR1 modulates tumourigenesis and paclitaxel response in MKN45 gastric cancer cells. Cell Cycle.

[36] Andonegui-Elguera MA, Cáceres-Gutiérrez RE, Luna-Maldonado F, López-Saavedra A, Díaz-Chávez J, Cisneros-Soberanis F, Prada D, Mendoza-Pérez J, Herrera LA (2016). BUB1 and SURVIVIN proteins are not degraded after a prolonged mitosis and accumulate in the nuclei of HCT116 cells. Cell Death Discov.

[37] Lee EA, Keutmann MK, Dowling ML, Harris E, Chan G, Kao GD (2004). Inactivation of the mitotic checkpoint as a determinant of the efficacy of microtubule-targeted drugs in killing human cancer cells. Mol Cancer Ther.

[38] Chen F, Wu P, Hu H, Tian D, Jiang N, Wu C (2018). Protein kinase TTK promotes proliferation and migration and mediates epithelial–mesenchymal transition in human bladder cancer cells. Int J Clin Exp Pathol.

[39] Qin X, Yan L, Zhao X, Li C, Fu Y (2012). microRNA-21 overexpression contributes to cell proliferation by targeting PTEN in endometrioid endometrial cancer. Oncol Lett.

[40] Sato K, Miyamoto M, Takano M, Tsuda H (2021). MicroRNA-21 expression in cancer cells is an independent biomarker of progression-free survival of endometrioid endometrial carcinoma. Virchows Arch.

[41] Lei X, Zhang M, Guan B, Chen Q, Dong Z, Wang C (2021). Identification of hub genes associated with prognosis, diagnosis, immune infiltration and therapeutic drug in liver cancer by integrated analysis. Hum Genom.

[42] Li Y, Zhou X, Liu J, Yin Y, Yuan X, Yang R, Wang Q, Ji J, He Q (2020). Differentially expressed genes and key molecules of BRCA1/2-mutant breast cancer: evidence from bioinformatics analyses. PeerJ.

[43] Chen J, Wu R, Xuan Y, Jiang M, Zeng Y (2020). Bioinformatics analysis and experimental validation of TTK as a biomarker for prognosis in non-small cell lung cancer. Biosci Rep.

[44] Gao YH, Qu SS, Cao LQ, Yao M (2022). TTK predicts triple positive breast cancer prognosis and regulates tumor proliferation and invasion. Neoplasma.

[45] Lu N, Ren L (2021). TTK (threonine tyrosine kinase) regulates the malignant behaviors of cancer cells and is regulated by microRNA-582-5p in ovarian cancer. Bioengineered.

[46] Talukdar S, Chang Z, Winterhoff B, Starr TK (2021). Single-cell RNA sequencing of ovarian cancer: promises and challenges. Adv Exp Med Biol.

[47] Yu Z, Zhang J, Zhang Q, Wei S, Shi R, Zhao R, An L, Grose R, Feng D, Wang H (2022). Single-cell sequencing reveals the heterogeneity and intratumoral crosstalk in human endometrial cancer. Cell Prolif.

[48] Olbrecht S, Busschaert P, Qian J, Vanderstichele A, Loverix L, Van Gorp T, Van Nieuwenhuysen E, Han S, Van den Broeck A, Coosemans A, Van Rompuy AS, Lambrechts D, Vergote I (2021). High-grade serous tubo-ovarian cancer refined with single-cell RNA sequencing: specific cell subtypes influence survival and determine molecular subtype classification. Genome Med.

[49] Panchy N, Azeredo-Tseng C, Luo M, Randall N, Hong T (2020). Integrative transcriptomic analysis reveals a multiphasic epithelial–mesenchymal spectrum in cancer and non-tumorigenic cells. Front Oncol.

[50] Saitoh M (2018). Involvement of partial EMT in cancer progression. J Biochem.

[51] Tseng JH, Bisogna M, Hoang LN, Olvera N, Rodriguez-Aguayo C, Lopez-Berestein G, Sood AK, Levine DA, Jelinic P (2017). miR-200c-driven mesenchymal-to-epithelial transition is a therapeutic target in uterine carcinosarcomas. Sci Rep.

[52] Bouziyane A, Lamsisi M, Benaguida H, Benhessou M, El Kerroumi M, Ennaji MM (2021). Diagnostic value of microRNA 21 in endometrial cancer and benign lesions and its differential expression with clinicopathological parameters. Microrna.

[53] Wang N, Zhang CQ, He JH, Duan XF, Wang YY, Ji X, Zang WQ, Li M, Ma YY, Wang T, Zhao GQ (2013). MiR-21 down-regulation suppresses cell growth, invasion and induces cell apoptosis by targeting FASL, TIMP3, and RECK genes in esophageal carcinoma. Dig Dis Sci.

[54] Martin del Campo SE, Latchana N, Levine KM, Grignol VP, Fairchild ET, Jaime-Ramirez AC, Dao TV, Karpa VI, Carson M, Ganju A, Chan AN, Carson WE (2015). MiR-21 enhances melanoma invasiveness via inhibition of tissue inhibitor of metalloproteinases 3 expression: in vivo effects of MiR-21 inhibitor. PLoS ONE.

[55] Zhang Z, Wang J, Wang X, Song W, Shi Y, Zhang L (2018). MicroRNA-21 promotes proliferation, migration, and invasion of cervical cancer through targeting TIMP3. Arch Gynecol Obstet.

[56] Li W, Song YY, Rao T, Yu WM, Ruan Y, Ning JZ, Yao XB, Yang SY, Cheng F (2022). CircCSNK1G3 up-regulates miR-181b to promote growth and metastasis via TIMP3-mediated epithelial to mesenchymal transitions in renal cell carcinoma. J Cell Mol Med.

[57] Chen J, Zhou C, Li J, Xiang X, Zhang L, Deng J, Xiong J (2018). miR-21-5p confers doxorubicin resistance in gastric cancer cells by targeting PTEN and TIMP3. Int J Mol Med.

[58] Qin WX, Shi Y, Zhu D, Li YP, Chen YH, Cui J, Cui GY, Pan JX, Ren ZY (2020). EZH2-mediated H3K27me3 enrichment on the lncRNA MEG3 promoter regulates the growth and metastasis of glioma cells by regulating miR-21-3p. Eur Rev Med Pharmacol Sci.

[59] Frezzetti D, De Menna M, Zoppoli P, Guerra C, Ferraro A, Bello AM, De Luca P, Calabrese C, Fusco A, Ceccarelli M, Zollo M, Barbacid M, Di Lauro R, De Vita G (2011). Upregulation of miR-21 by Ras in vivo and its role in tumor growth. Oncogene.

[60] Li Z, Yang L, Liu X, Nie Z, Luo J (2018). Long noncoding RNA MEG3 inhibits proliferation of chronic myeloid leukemia cells by sponging microRNA21. Biomed Pharmacother.

[61] Xia H, Zhang W, Zhang B, Zhao Y, Zhao Y, Li S, Liu Y (2017). miR-21 modulates the effect of EZH2 on the biological behavior of human lung cancer stem cells in vitro. Oncotarget.

[62] Mason JM, Wei X, Fletcher GC, Kiarash R, Brokx R, Hodgson R, Beletskaya I, Bray MR, Mak TW (2017). Functional characterization of CFI-402257, a potent and selective Mps1/TTK kinase inhibitor, for the treatment of cancer. Proc Natl Acad Sci USA.

[63] Sugimoto Y, Sawant DB, Fisk HA, Mao L, Li C, Chettiar S, Li PK, Darby MV, Brueggemeier RW (2017). Novel pyrrolopyrimidines as Mps1/TTK kinase inhibitors for breast cancer. Bioorg Med Chem.

[64] Yu J, Gao G, Wei X, Wang Y (2022). TTK protein kinase promotes temozolomide resistance through inducing autophagy in glioblastoma. BMC Cancer.

[65] Tannous BA, Kerami M, Van der Stoop PM, Kwiatkowski N, Wang J, Zhou W, Kessler AF, Lewandrowski G, Hiddingh L, Sol N, Lagerweij T, Wedekind L, Niers JM, Barazas M, Nilsson RJ, Geerts D, De Witt Hamer PC, Hagemann C, Vandertop WP, Van Tellingen O, Noske DP, Gray NS, Würdinger T (2013). Effects of the selective MPS1 inhibitor MPS1-IN-3 on glioblastoma sensitivity to antimitotic drugs. J Natl Cancer Inst.

[66] Chandler BC, Moubadder L, Ritter CL, Liu M, Cameron M, Wilder-Romans K, Zhang A, Pesch AM, Michmerhuizen AR, Hirsh N, Androsiglio M, Ward T, Olsen E, Niknafs YS, Merajver S, Thomas DG, Brown PH, Lawrence TS, Nyati S, Pierce LJ, Chinnaiyan A, Speers C (2020). TTK inhibition radiosensitizes basal-like breast cancer through impaired homologous recombination. J Clin Invest.

