# Supplementary material for: Integrated multi-omics analyses and functional validation reveal TTK as a novel EMT activator for endometrial cancer
Source: J Transl Med. 2023 Feb 25;21:151. doi: 10.1186/s12967-023-03998-8 (PMC9960418; doi:10.1186/s12967-023-03998-8)
Supplement: Supplementary file 1 — Additional file 1: Figure S1. Expression of specific genes in EC. Figure S2. Differences in SMG and CNV associated with dysregulated TTK gene expression in EC. Figure S3. Extensive genomic changes of TTK gene in human cancers. Figure S4. Epigenetic differences associated with TTK dysregulation in EC. Figure S5. EMT-related genes expression across major cell types. Figure S6. Pharmacologic inhibition of TTK mimicked the effects of TTK silencing on EC cells. [file 12967_2023_3998_MOESM1_ESM.docx]

Additional file 1

Integrated multi-omics analyses and functional validation reveal TTK as a novel EMT activator for endometrial cancer

Yu Miao^1,2,†^ (miaoyu19@mails.ucas.ac.cn), Yosuke Konno^3,†^ (konsuke013@gmail.com), Baojin Wang^4,†^ (307797362@qq.com), Lin Zhu^2^ ([zhulin1@genomics.cn](mailto:zhulin1@genomics.cn)), Tianyue Zhai^3^ (zhaitianyue@gmail.com), Kei Ihira^3^ (ihey0610@huhp.hokudai.ac.jp), Noriko Kobayashi^3^ (norikingyo@med.hokudai.ac.jp), Hidemichi Watari^3^ (watarih@med.hokudai.ac.jp), Xin Jin^2^ (jinxin@genomics.cn),Junming Yue^5,6,^* ([jyue@uthsc.edu](mailto:jyue@uthsc.edu)), Peixin Dong^3,^* ([dpx1cn@gmail.com](mailto:dpx1cn@gmail.com)) and Mingyan Fang^2,7,^* (angmingyan@genomics.cn)

1. College of Life Sciences, University of Chinese Academy of Sciences, Beijing 100049, China
2. BGI-Shenzhen, Shenzhen 518083, China
3. Department of Obstetrics and Gynecology, Hokkaido University School of Medicine, Hokkaido University, Sapporo 060-8638, Japan
4. Department of Gynecology and Obstetrics, Third Affiliated Hospital, Zhengzhou University, Zhengzhou 450052, China
5. Department of Pathology and Laboratory Medicine, University of Tennessee Health Science Center, Memphis, TN 38163, USA
6. Center for Cancer Research, University of Tennessee Health Science Center, Memphis, TN 38163, USA
7. BGI Research Asia-Pacific, BGI, Singapore 138567, Singapore

^†^ Yu Miao, Yosuke Konno and Baojin Wang are co-first authors and contributed equally to this work.

* Correspondence: Mingyan Fang (fangmingyan@genomics.cn), Peixin Dong (dpx1cn@gmail.com) and Junming Yue ([jyue@uthsc.edu](mailto:jyue@uthsc.edu))

**Figure legends**

**
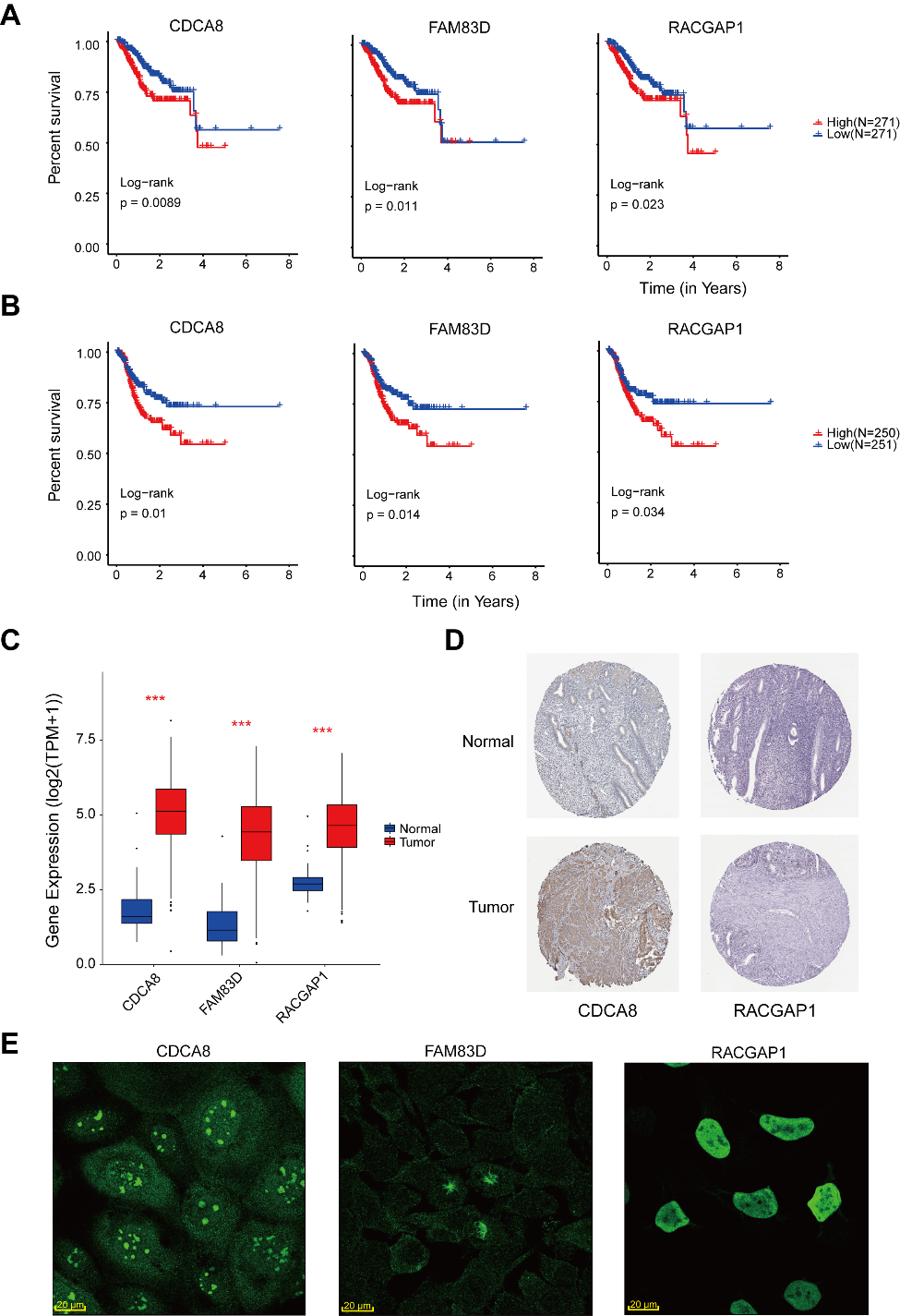
**

Figure S1: Expression of specific genes in EC

(A) Relationship between indicated gene expression and overall survival. (B) The relationship between indicated gene expression and disease-free survival. (C) Differential gene expression of three genes (*CDCA8, FAM83D,* and *RACGAP1*) in EC and normal tissues. (D) An immunohistochemical picture from the HPA database demonstrates high CDCA8 and FAM83D protein expression in EC tissues than in adjacent normal tissues. (E) Results of subcellular localization of CDCA8, FAM83D, and RACGAP1 protein, green indicates target protein, blue indicates nucleus and red indicates microtubule. Scale bars = 20 μm. **P* < 0.05, ***P* < 0.01, ****P* < 0.001 (Wilcoxon test).


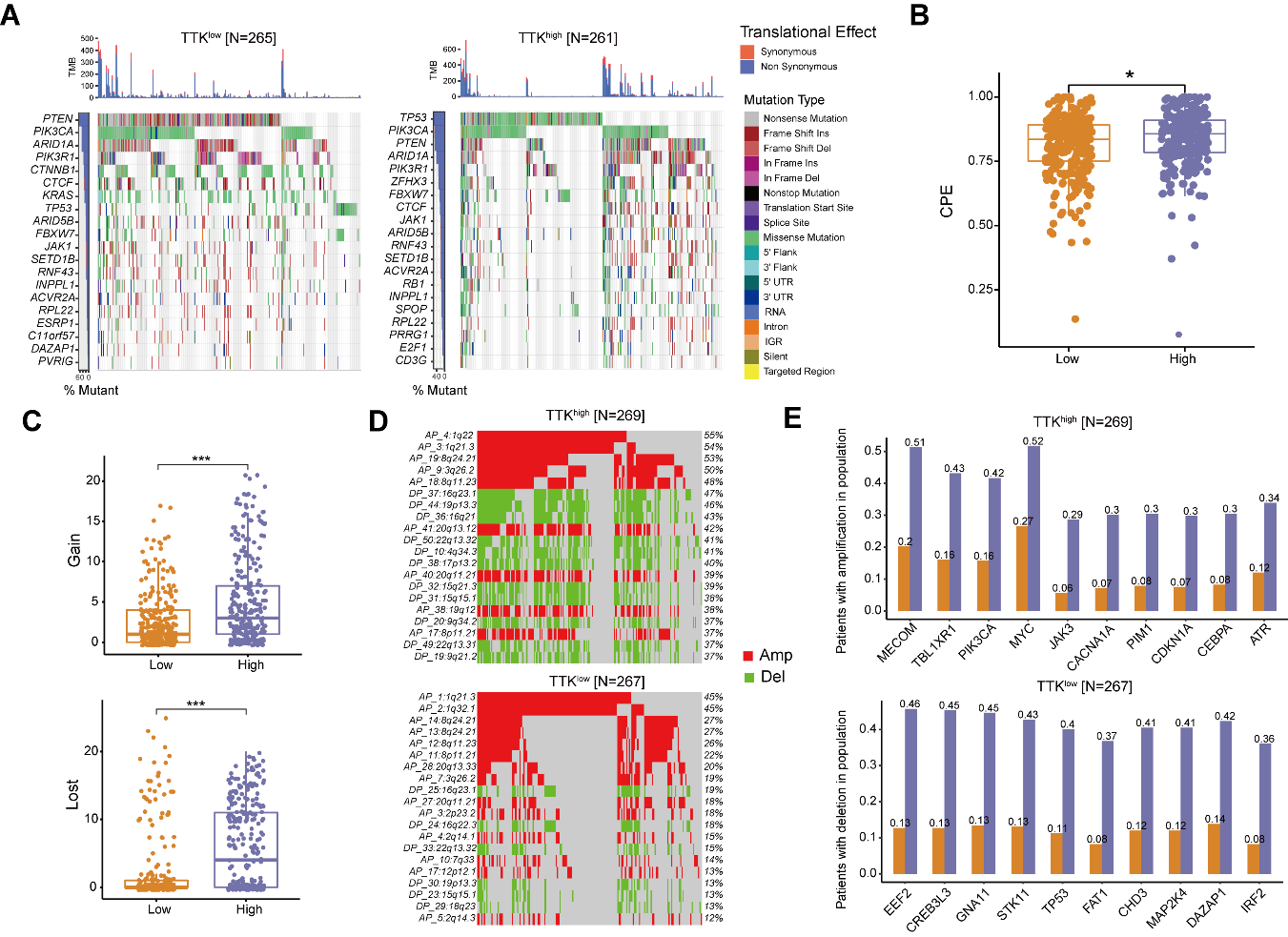


Figure S2: Differences in SMG and CNV associated with dysregulated TTK gene expression in EC

(A) The left and right plots show the top 20 SMG in the patients of the *TTK*^low^ (left) and *TTK^high^* groups (right), respectively. (B) Differences in tumor purity between the TTK^high^ and TTK^low^ groups. (C) Differences in the number of CNVs between the TTK^high^ and TTK^low^ groups. (D) Top20 CNVs with high-frequency variation in the TTK^high^ and TTK^low^ groups in EC. (E) Box plots demonstrate the difference in population frequencies of amplification and deletion of driver genes between the two categories of TTK^high^ and TTK^low^ groups. **P* < 0.05, ***P* < 0.01, ***P < 0.001 (Wilcoxon test).


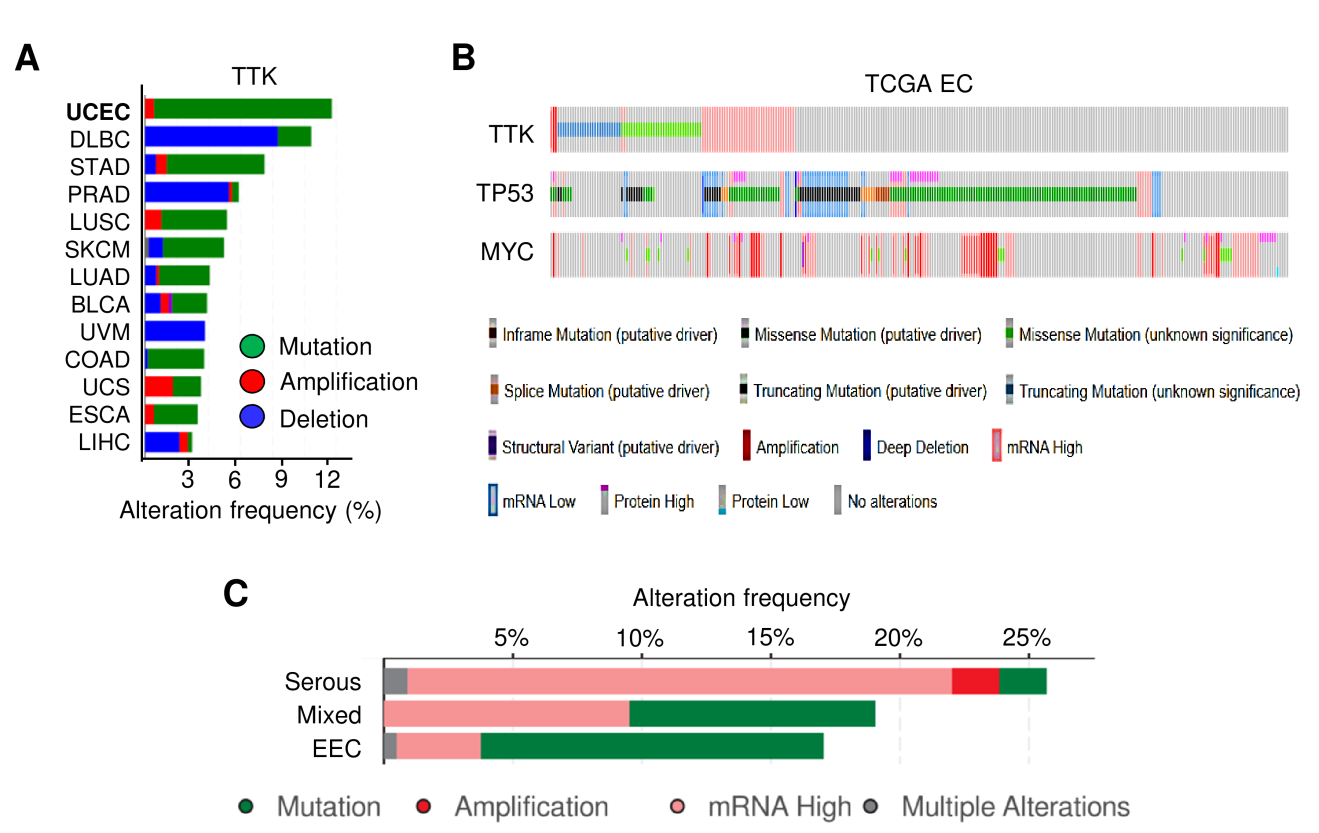


Figure S3: Extensive genomic changes of *TTK* gene in human cancers

(A) Cancer types summary showing genetic alterations in the *TTK* gene across TCGA cancer studies (cBioPortal). (B) An Oncoprint demonstrating the genetic changes in the *TTK*, *TP53,* and *MYC* genes in TCGA EC tissues (cBioPortal). (C) Proportions of serous, EEC, or mixed ECs with different genetic changes in the *TTK* gene (cBioPortal).


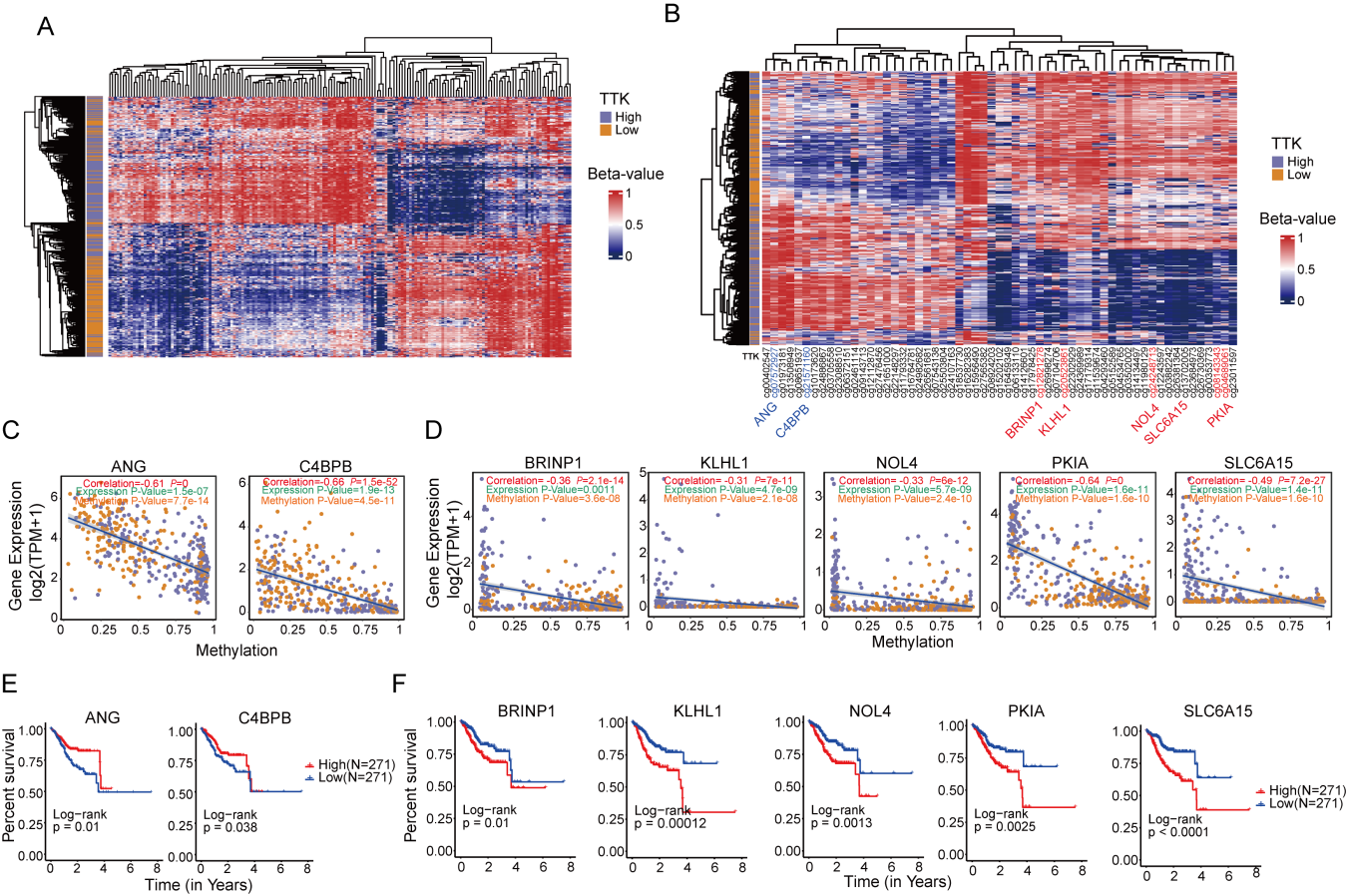


Figure S4: Epigenetic differences associated with TTK dysregulation in EC

(A) DMGs between the TTK^high^ and TTK^low^ groups. (B) DMGs in plot A located in the promoter regions of genes; Genes marked in red are upregulated genes with hypomethylation; Genes marked in blue are down-regulated genes with hypermethylation. (C and D) Correlation between DMG methylation levels and gene expression in the promoter regions of down-regulated genes with hypermethylation (C) and upregulated genes with hypomethylation (D). Brown dots indicate samples from the TTK^low^ group; Purple dots indicate samples from the TTK^high^ group. The red font indicates correlation coefficients and significance. The green font shows differences between gene expressions. The orange font shows the difference between methylation levels. (E and F) Relationship between down-regulated genes with hypermethylation (E) and upregulated genes with hypomethylation (F) expression and overall patient survival. Survival curves were created and analyzed using the log-rank tests.


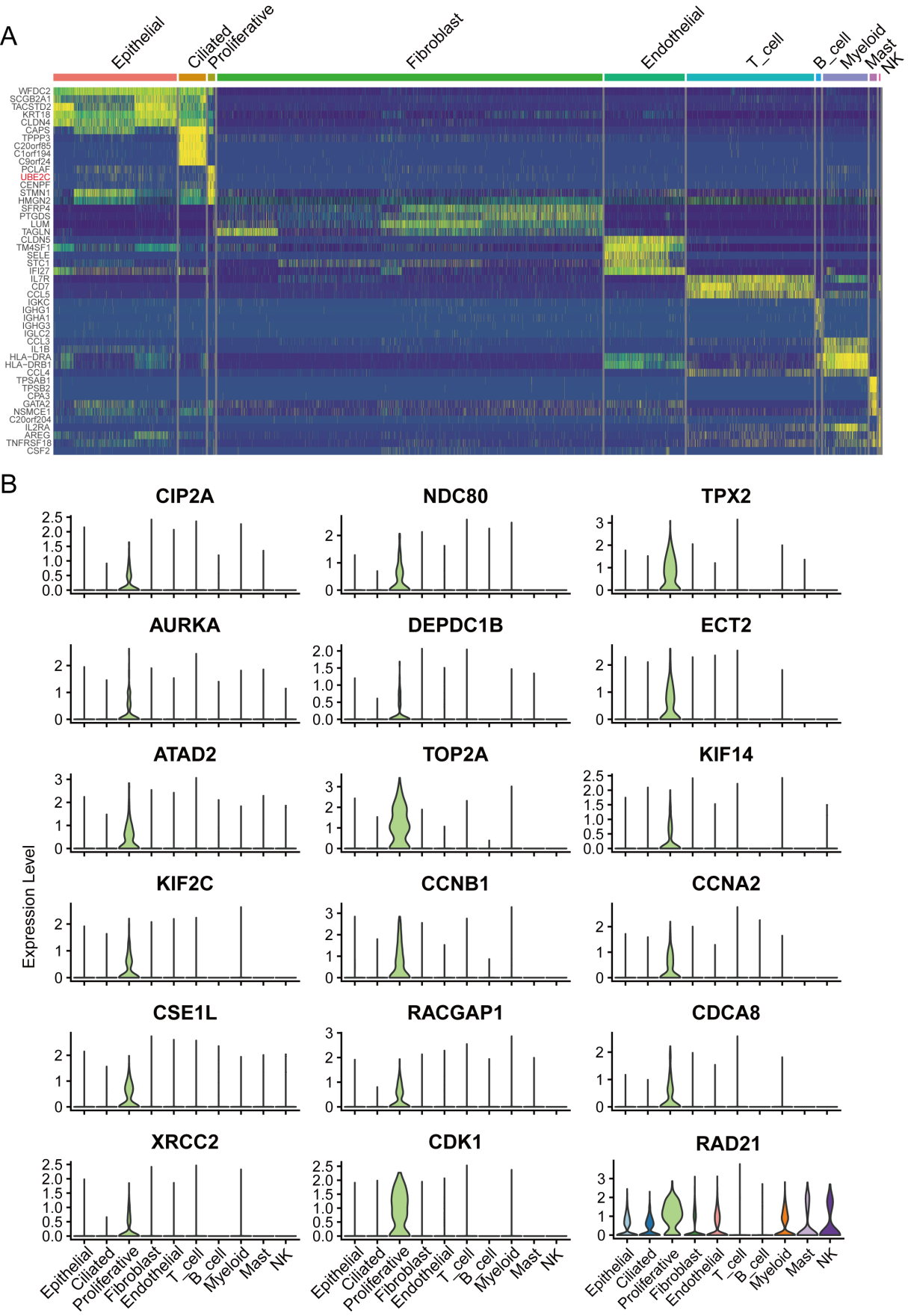


Figure S5: EMT-related genes expression across major cell types

(A) Heatmap displays the genes highly expressed in all cell subpopulations. (B) Violin plots show the expression of EMT-related genes with a significant positive connection to *TTK* across major cell types.

**
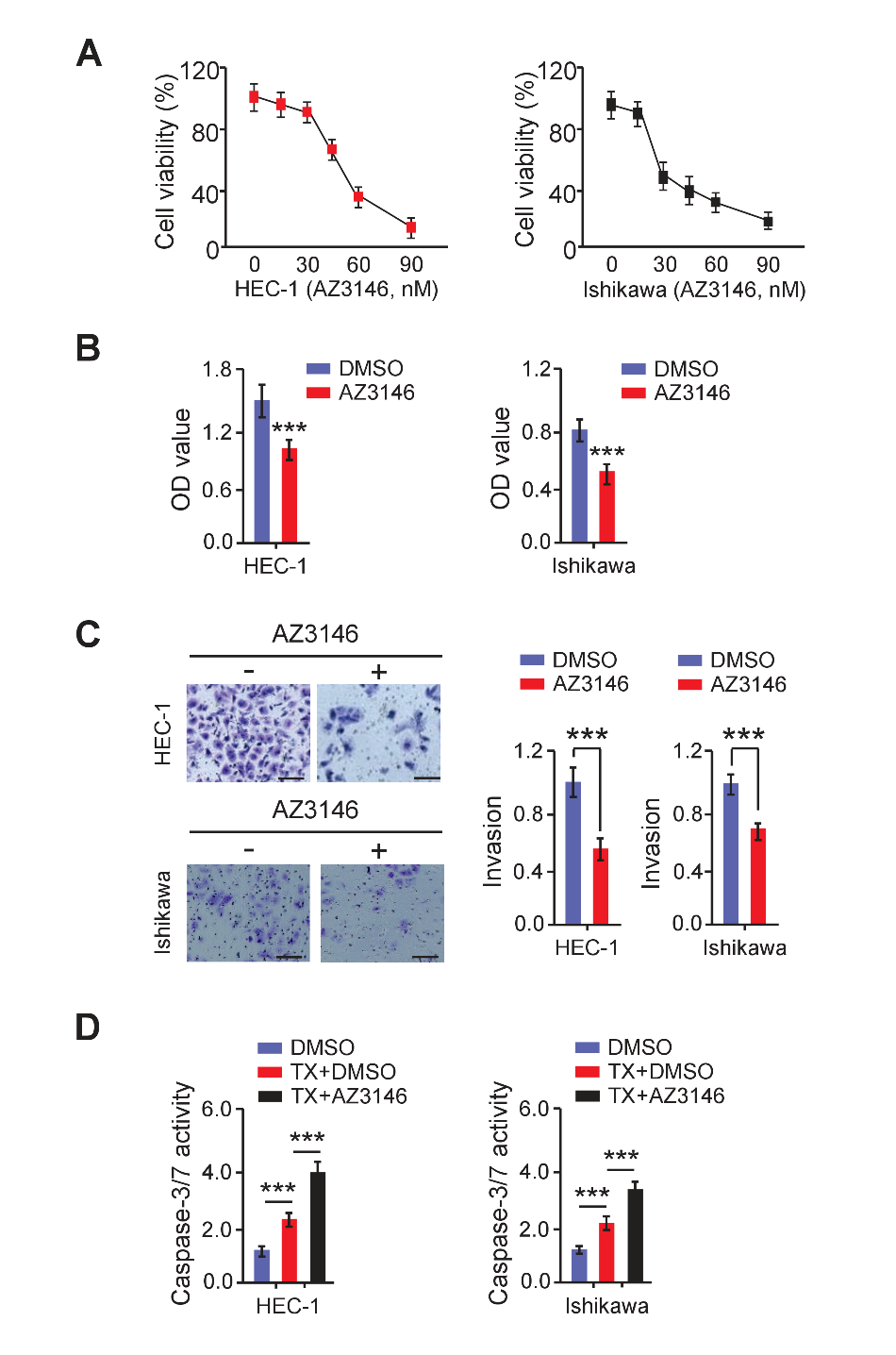
**

**Figure S6: Pharmacologic inhibition of TTK mimicked the effects of TTK silencing on EC cells.**

(A) The cytotoxicity of the TTK inhibitor AZ3146 was evaluated using MTT assays. (B and C) The impact of AZ3146 on the growth (B) and invasion (C) of EC cells were examined using CCK-8 and cell invasion assays, respectively. Scale bars = 50 μm. ****P* < 0.001, by Student’s *t-*tests. (D) EC cells were exposed to TX (50 nM for HEC-1 cells and 10 nM for Ishikawa cells) with or without AZ3146 for 24 hours. Cell apoptosis was measured using Caspase-Glo 3/7 assays. ****P* < 0.001, by Student’s *t-*tests.
